# Clinical and preclinical therapeutic outcome metrics for *USH2A*-related disease

**DOI:** 10.1093/hmg/ddaa004

**Published:** 2020-01-30

**Authors:** Maria Toms, Adam M Dubis, Erik de Vrieze, Dhani Tracey-White, Andreas Mitsios, Matthew Hayes, Sanne Broekman, Sarah Baxendale, Nattawan Utoomprurkporn, Doris Bamiou, Maria Bitner-Glindzicz, Andrew R Webster, Erwin Van Wijk, Mariya Moosajee

**Affiliations:** 1 UCL Institute of Ophthalmology, University College London, London EC1V 9EL, UK; 2 Moorfields Eye Hospital NHS Foundation Trust, London EC1V 2PD, UK; 3 Department of Otorhinolaryngology, Donders Institute for Brain, Cognition and Behaviour, Radboud University Medical Center, Nijmegen 6525 HR, The Netherlands; 4 Bateson Centre and Department of Biomedical Science, University of Sheffield, Sheffield S10 2TN, UK; 5 UCL Ear Institute, University College London, London WC1X 8EE, UK; 6 Faculty of Medicine, Chulalongkorn University, Bangkok 10330, Thailand; 7 Great Ormond Street Hospital for Children NHS Foundation Trust, London WC1N 3JH, UK

## Abstract

*USH2A* variants are the most common cause of Usher syndrome type 2, characterized by congenital sensorineural hearing loss and retinitis pigmentosa (RP), and also contribute to autosomal recessive non-syndromic RP. Several treatment strategies are under development; however, sensitive clinical trial endpoint metrics to determine therapeutic efficacy have not been identified. In the present study, we have performed longitudinal retrospective examination of the retinal and auditory symptoms in (i) 56 biallelic molecularly confirmed *USH2A* patients and (ii) *ush2a* mutant zebrafish to identify metrics for the evaluation of future clinical trials and rapid preclinical screening studies. The patient cohort showed a statistically significant correlation between age and both rate of constriction for the ellipsoid zone length and hyperautofluorescent outer retinal ring area. Visual acuity and pure tone audiograms are not suitable outcome measures. Retinal examination of the novel *ush2a^u507^* zebrafish mutant revealed a slowly progressive degeneration of predominantly rods, accompanied by rhodopsin and blue cone opsin mislocalization from 6 to 12 months of age with lysosome-like structures observed in the photoreceptors. This was further evaluated in the *ush2a^rmc^* zebrafish model, which revealed similar changes in photopigment mislocalization with elevated autophagy levels at 6 days post fertilization, indicating a more severe genotype-phenotype correlation and providing evidence of new insights into the pathophysiology underlying *USH2A*-retinal disease.

## Introduction

Usher syndrome, characterized by combined sensorineural hearing loss and retinitis pigmentosa (RP), is the most common cause of deaf-blindness worldwide (1,2). Three clinical types (Usher syndrome types 1, 2 and 3) can be distinguished based on the severity and progression of the hearing loss and presence or absence of vestibular dysfunction, with type 1 being the most severe and type 2 (USH2) being the most frequent (2). Mutations in *USH2A* (MIM #608400) are the most frequent cause of Usher syndrome, accounting for up to 85% of USH2 cases, as well as causing up to 23% of non-syndromic autosomal recessive RP (3). There is a broad *USH2A* mutation spectrum with the two most common variants located in exon 13 (i) c.2299delG, p.(Glu767fs) and (ii) c.2276G>T, p.(Cys759Phe) (4–6). *USH2A* encodes usherin, a 5202 amino acid transmembrane protein with domains common to extracellular matrix proteins and cell adhesion proteins, including several predicted laminin domains and fibronectin III repeats. A short secreted isoform of 1546 amino acid in size is also expressed in various tissues, including intestine and testis, but the full-length protein is thought to be predominantly expressed in the retina and cochlea (7,8).

In the retina, usherin co-localizes with the two other known USH2-related proteins, adhesion G protein-coupled receptor V1 (ADGRV1) and whirlin, at the periciliary membrane (8–10). This structure is thought to be involved in docking and fusion of transport vesicles that contain cargo bound for the photoreceptor connecting cilium and outer segment. Usherin is also known to interact with other Usher proteins, including harmonin (USH1C), myosin VIIa (USH1B) and SANS (USH1G) in the photoreceptors (9,11–13). In the mammalian cochlea, an USH2 complex consisting of usherin, ADGRV1 and whirlin, forms the transient ankle links between adjacent stereocilia of the developing hair cell bundles (14,15). These links are thought to play a role in regulating stereociliary growth, differentiation and organization during postnatal development.

USH2 is characterized by moderate-to-severe congenital sensorineural hearing loss with normal vestibular function. Usher syndrome-associated visual impairment is the result of RP and presents with night blindness (nyctalopia) and loss of peripheral visual field caused by rod photoreceptor degeneration, progressing to involve cones leading to central vision loss, often resulting in legal blindness (16). There is significant intra- and inter-familial phenotypic variability, which has recently also been reflected in *ush2a* zebrafish disease models (10,17). The full-length zebrafish *ush2a* gene (transcript ID. ENSDART00000086201.5) consists of 72 exons (15 708 bp of coding sequence) and encodes the usherin protein, which shares 52% identity and 68% similarity to the human orthologue. CRISPR/Cas9 generated mutants *ush2a^rmc1^* [c.2337_2342delinsAC; p.(Cys780Glnfs^*^32) in exon 13] and *ush2a^b1245^* [c.15520_15523delinsTG; p.(Ala5174fs^*^) in exon 71] revealed defective electroretinogram (ERG) responses at 5 days post fertilization (dpf) and photoreceptor cell death following exposure to constant bright light (3000 lux) for 72 h at 8 dpf (10). However, examination at adult ages was limited and did not reveal clear progressive abnormalities. In contrast, a TALENs-generated *ush2a^hzu6^* zebrafish [c.136_152delGCCCCTCAGGGCAACTT; p.(Ala46Profs^*^10), exon 1] was described as showing early defects in ERG and acoustic startle responses with a progressive rod-cone degeneration in the adult fish, apparent from 12 months due to shortening of the photoreceptor outer segments (17). Levels of rod-specific proteins rhodopsin, GNAT1 and GNB1 were reduced from 7 months.

While hearing loss in patients can be ameliorated with the use of hearing aids or cochlear implants, there is currently no treatment available to prevent the RP-induced vision loss. Several therapies are in preclinical development including small molecule drugs (18), CRISPR/Cas9-based genome editing (19) and antisense oligonucleotides (20,21). Little is known about suitable outcome measures for USH2 prospective clinical trials due to limited clinical longitudinal natural history studies in the literature. In this study, we have explored the long-term *USH2A* pathophysiology in humans and examined the molecular defects underlying *USH2A*-associated retinal degeneration in a novel zebrafish mutant [*ush2a^u507^*; c.2131_2203+73delinsCGGCGG; p.(Ala711fs^*^), exon 12; transcript ID: XM_009293147.3] and the previously published *ush2a^rmc1^* mutant, to further improve our understanding of the molecular pathology underlying the disease and to identify potential outcome measures for assaying a response to emerging therapeutics in both preclinical and clinical studies.

## Results

### Molecular and clinical characteristics of patients

**Figure 1:**
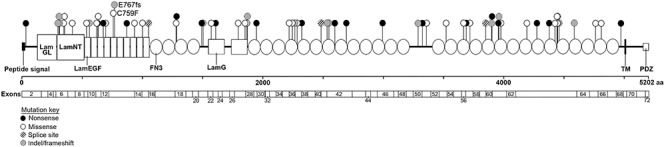
Position and type of *USH2A* variants in participating patients. Schematic diagram of the usherin protein domain architecture and *USH2A* exon structure. The genetic position and type of mutation of the identified *USH2A* variants in the patients within this study are presented as lollypops at the corresponding location in the usherin protein. Black labels correspond to nonsense mutations, white labels correspond to missense variants, diagonal lines correspond to splice site variants and grey labels correspond to insertion and deletion frameshift changes*.* LamGL, laminin globular-like domain; LamNT, N-terminal laminin domain; LamEGF, laminin-type epidermal growth factor-like domains; FN3, fibronectin III domains; TM, transmembrane domain; PDZ, PDZ-binding domain.

**Figure 2:**
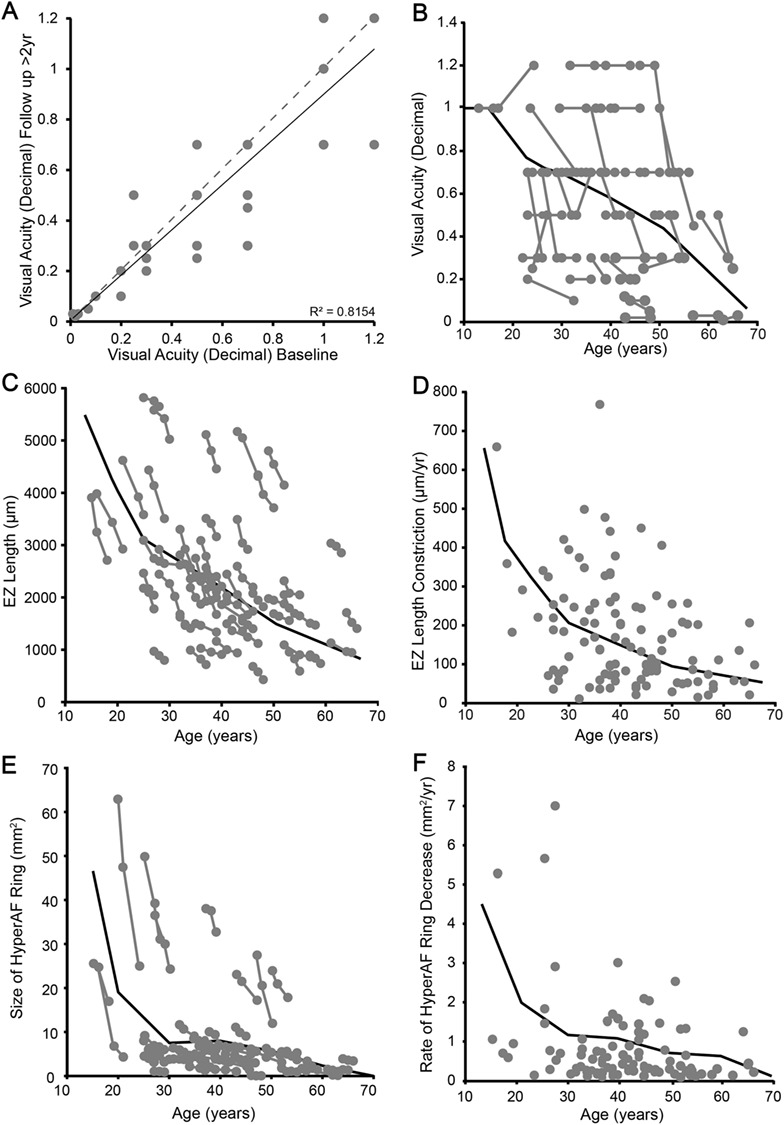
Visual acuity, EZ and hyperautofluorescent (hyperAF) ring measurements with age in *USH2A* patients. (**A**) and (**B**) Change in visual acuity over the observation period. (A) illustrates the change in BCVA between baseline and final follow up. The grey dashed line represents ‘no change’ while the black correlation line illustrates the subtle change. The measurement of each patient at each visit is depicted as a function of age in (B). (**C**) Correlation between EZ length and age. (**D**) EZ constriction rate (change in size divided by time between visits) as a function of age. Both (C) and (D) illustrate that the degeneration starts rapidly with later, slower decline. (**E**) and (**F**) illustrate the same changes in hyperAF ring area.

To investigate clinical trial outcome measures for *USH2A*-related retinal disease, patients with molecularly confirmed biallelic *USH2A* mutations (*n* = 56, average age 40 years, range 15–66 at first visit) were retrospectively assessed. Collectively they displayed 67 distinct variants including 19 nonsense variants, 29 missense variants, 16 frameshift-inducing indels and 3 variants affecting pre-mRNA splicing (Fig. 1 and Supplementary Material, Table S1). Among these variants, the following were previously unreported: c.5555A>G p.(His1852Arg), c.7789A>T p.(Lys2597^*^), c.7883dupC p.(Pro2628Profs^*^7) and c.11156G>T p.(Arg3719Leu). There was a strong agreement in best-corrected visual acuities (BCVA) between eyes with a net bias of 0.05 decimal acuity units and a 95% confidence interval of +0.41 to −0.37 (Fig. 2A and B). There was a strong statistical correlation between acuity at baseline and 2 year follow-up (*r*^2^ = 0.815, *P* < 0.0001) with little change in BCVA. Only 23 of 56 patients had any change in visual acuity between first and last visits (range: +0.25 to −0.5 decimal units).

### Longitudinal changes in hyperautofluorescent ring area and ellipsoid zone length

All patients displayed a hyperautofluorescent (hyperAF) outer ring phenotype, better described as elliptical rather than circular (*r*^2^ = 0.99 versus 0.95), with the vertical diameter on average 363 μm shorter than horizontal. At baseline, 53/56 patients had the classic dual hyper/hypoAF rings (the remaining four patients had only hyperAF rings at baseline); this decreased to 52 patients at time point 1 and to 48 patients by time point 2. The loss of the hypoAF ring phenotype occurred in subjects with the least ring area at baseline and likely correlated with insufficient residual retina to produce the dual ring phenotype. The hyperAF ring was the more stable and repeatable metric, and thus followed for longitudinal trends. Size of the hyperAF ring was biphasic with a very rapid decline in area over the first ~ 30 years of life, then becoming much less steep in the following decades of life. For instance, the average ring area for <30 years old was 20.1 mm^2^ (range 63.13–1.14 mm^2^) compared to 6.96 mm^2^ (range 38.26–0.18 mm^2^) for those >30 years of age. The reduction in hyperAF area is highly variable across all ages (Fig. 2E). The average rate of hyperAF constriction was 1.42 mm^2^ (25.32–0.03 mm^2^) for the first follow-up period and slowed only slightly to 1.01 mm^2^ (range 6.06–0.01 mm^2^) for the second follow-up period. The rates were only weakly correlated between the two time points (*r*^2^ = 0.22, *P* = 0.001) (Fig. 2F).

Ellipsoid zone (EZ) length compared to age follows a non-linear function (Fig. 2C), which is not well described by logarithmic or multiphasic declines as there is no clear inflection point. The average EZ length for the under 30 cohort was 3534 μm (±1449 μm SD), while the over 50 subjects had a residual EZ length of 1653 (±679 μm SD). However, discrete analysis shows that the decline is faster at younger rather than at older ages linked to the finite amount of residual retina. The rate of constriction follows a similar pattern to residual length, although there is a clear inflection point at about 30 years of age (Fig. 2D). For instance, average degeneration rate in subjects under 30 was 204 μm/year (range of 36–659 μm/year). The >50 years of age cohort showed the average constriction rate dropped to 112 μm/year (range 48–257 μm/year). Mean change in retinal thickness was 7.7 μm/year (range: +151.9 to −98.1 μm/year), but measurements were confounded by macular edema and therefore not consistent with the retinal degeneration (Supplementary Material, Fig. S2).

**Figure 3:**
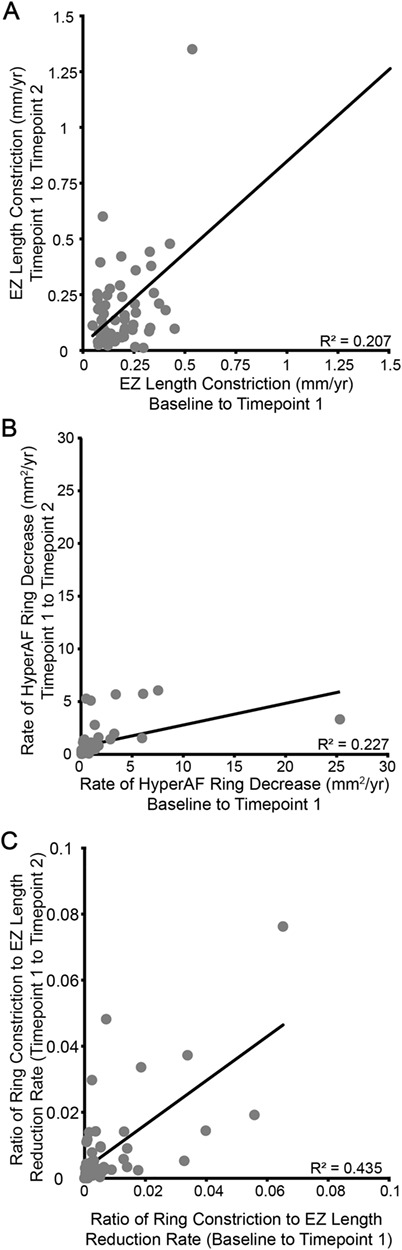
Inter-relatedness of progression between metrics. While there is a strong correlation between the rate of change observed in the first and second observation periods for both EZ length constriction (**A**) and hyperautofluorescence (hyperAF) ring constriction (**B**), neither correlation line describes the data well (*r*^2^ = 0.207 and 0.227 respectively). The ratio of EZ length and hyperAF ring constriction accounts for a much greater share of the variability (**C**, *r*^2^ = 0.4355).

In order to design a trial for an inherited retinal disease using systemic-delivered therapies, such as small molecule drug ataluren (Translarna™), a factor which predicts future progression must be developed. This is in contrast to surgical intervention for conventional viral gene therapies in which the fellow eye can be used as an internal control. When we evaluate the prediction of follow-up period 1 versus 2 for the EZ length, the values are significantly correlated (*r*^2^ = 0.207, *P* = 0.001) (Fig. 3A). This improves if hyperAF ring constriction rate is used (*r*^2^ = 0.227, *P* = 0.0008) (Fig. 3B), but this can be absent in advanced cases (22). While both of these are statistically significant due to the number of patients in this study, neither correlation singularly accounts for the majority of variability in the data. Therefore, more complex relationships need to be evaluated in order to attempt to design a study for USH2. If a multimodal approach is adopted using the ratio of EZ and hyperAF rates, a highly significant relationship is seen (*r*^2^ = 0.44, *P* < 0.0001) (Fig. 3C).

### Degree and progression of hearing loss

**Figure 4:**
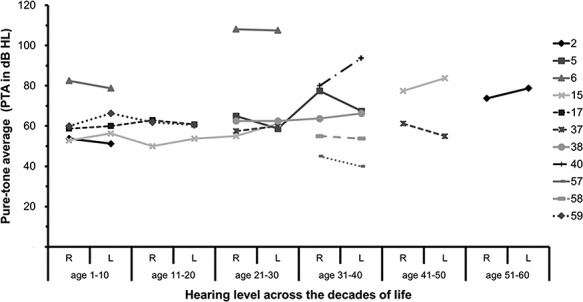
Degree and progression of hearing loss in *USH2A* patients. Pure tone average (PTA) measurements (decibel hearing loss, dB HL) from right (R) and left (L) ears of *USH2A* patients obtained within different decades of lifetime. Color key corresponds to patient number.

There were 11 patients with retrospectively available audiometric information (Supplementary Material, Table S2). All patients (except patient 6, who was progressive from severe to profound from first to third decade of life) had moderate to severe hearing loss when tested at any age with relatively stable hearing levels when the audiogram was repeated (Fig. 4). Audiometric configuration showed 10 patients had high frequencies hearing loss (i.e. more prominent hearing loss at frequencies above 2000 Hz). Patient 59 had a stable flat configuration of their hearing loss at all time points from their first to third decade. All patients had symmetrical hearing loss between both right and left ears.

No genotype–phenotype correlation could be ascertained due to compound heterozygosity and small numbers. Patient 6 with severe progressive hearing loss had compound heterozygous mutations: c.2299delG, p.(Glu767Serfs21^*^) and c.14911C>T, p.(Arg4971^*^). The variant c.2299delG was identified in seven other cases with non-progressive hearing loss, while the c.14911C>T variant (found only in this case) was not found to be significantly associated with progressive hearing loss (*P* = 0.25, Fischer’s exact test). Patient 59, the only case with the flat configuration of hearing loss had mutations at c.920_923dupGCCA (p.His308Glnfs) and c.3518C>A, p.(Ser1173^*^), which were not found in other patients.

### Reduced visual function in *ush2a*^*u507*^ zebrafish

**Figure 5:**
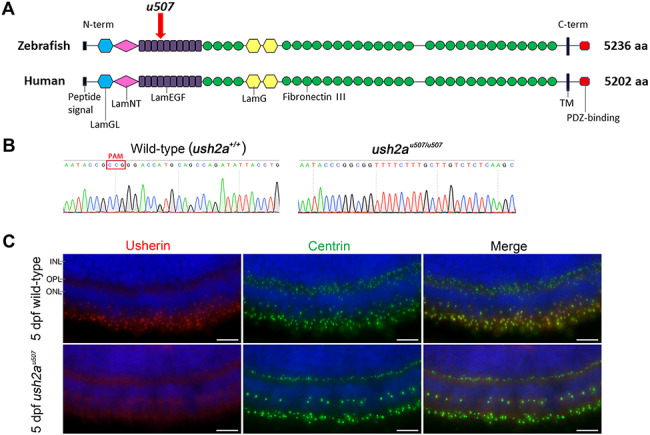
Generation of *ush2a^u507^*zebrafish. (**A**) Schematic representation of zebrafish and human usherin domain architecture. The *u507* mark on zebrafish usherin indicates the region targeted by CRISPR/Cas9. (**B**) Sanger sequencing traces showed the *ush2a* gene sequence change in homozygous *ush2a^u507^* mutant zebrafish (c.2131_2203 + 73delinsCGGCGG) at the CRISPR/Cas9 target site. (**C**) Anti-usherin-C (red) and anti-centrin (green) immunostaining of retinal sections from wt and *ush2a^u507^* larvae (5 dpf). DAPI nucleic acid stain was used as a counterstain (blue). PAM, protospacer adjacent motif. INL, inner nuclear layer; OPL, outer plexiform layer; ONL, outer nuclear layer; dpf, days post fertilization. Scale bar = 10 μm.

The CRISPR/Cas9 generated *ush2a^u507^* mutant line carried a 146 bp deletion in exon 12 and the following intron with a 6 bp insertion; c.2131_2203+73delinsCGGCGG, p.(Ala711fs), verified using Sanger sequencing (Fig. 5B). On transcript level, this deletion results in the exclusion of exon 12 from the *ush2a* mRNA, resulting in a frameshift and premature stop codon. Using an antibody directed against the C-terminus, usherin was shown to be expressed in the photoreceptors of 5 days post fertilization (dpf) wild-type (wt) zebrafish retina where it localized adjacent to the connecting cilium marker centrin (Fig. 5C). Usherin was not detected in the *ush2a^u507^* retina. RNAscope assay revealed notably reduced levels of *ush2a* mRNA in the adult *ush2a^u507^* photoreceptors (outer nuclear layer [ONL]) compared to wt, indicative of nonsense-mediated decay (Supplementary Material, Fig. S4).

**Figure 6:**
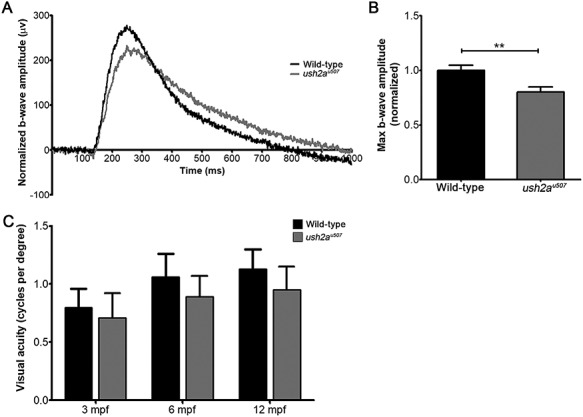
Visual function in *ush2a^u507^* zebrafish. (**A**) Normalized average b-wave amplitude of **ERG** responses recorded from wt and *ush2a^u507^* zebrafish at 5 days post fertilization (wt: *n* = 69, *ush2a^u507^*: *n* = 67). (**B**) Maximum b-wave amplitudes are plotted as bar graphs (mean ± SEM). (**C**) Visual acuity (cycles per degree, cpd) of wt and *ush2a^u507^* zebrafish at 3 months post fertilization (mpf), 6 mpf and 12 mpf, assessed using the optokinetic response assay (*n* = 4–5, mean ± SEM). ^*^^*^P < 0.001. Mann Whitney tests and two-way ANOVA were used to test for statistical significance in ERG and optokinetic response data, respectively.

To assess visual function in *ush2a^u507^* larvae, ERG responses were recorded from zebrafish at 5 dpf (Fig. 6A). The ERG maximum b-wave amplitudes were found to be significantly reduced in *ush2a^u507^* larvae as compared to wt controls (*P* < 0.01; wt *n* = 69, *ush2a^u507^ n* = 67) (Fig. 6B). Visual function was also tested in adult zebrafish using optokinetic response testing but was not found to be statistically significant at any age (Fig. 6C); at 3, 6 and 12 mpf, visual acuity was 0.71 ± 0.21, 0.89 ± 0.18 and 0.95 ± 0.20 cycles per degree (cpd), respectively, in *ush2a^u507^* compared to 0.80 ± 0.16, 1.06 ± 0.20 and 1.13 ± 0.17 cpd in wt (*n* ≥ 4).

No gross abnormalities were noted on retinal histology up to 6 months post fertilization (mpf), but a significant difference (*P* < 0.01) in the number of ONL nuclei in *ush2a^u507^* fish was seen at 12 mpf (80.1 ± 4.99) compared to age-matched wt fish (102.1 ± 8.29) (*n* = 5) (Supplementary Material, Fig. S5). Cell death assays showed a significant increase in apoptotic photoreceptor nuclei per retinal section with age in the *ush2a^u507^* retina (0.47 ± 0.15, 0.73 ± 0.07 and 1.87 ± 0.27 at 3, 6 and 12 mpf, respectively; *n* ≥ 13, *P* < 0.001), while apoptotic nuclei were only detected in the 12 mpf wt retina (0.22 ± 0.22; *n* = 10) (Supplementary Material, Fig. S5). Apoptosis levels were significantly elevated at 6 and 12 mpf in the mutant compared to wt (*P* < 0.05 and *P* < 0.01, respectively).

### Structure and function of the mechanosensory hair cells in *ush2a*^*u507*^ zebrafish

**Figure 7:**
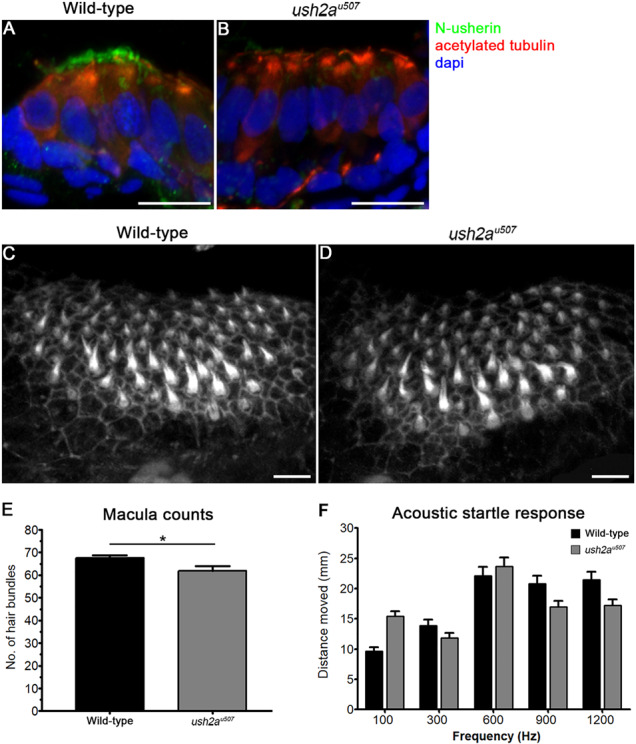
Auditory function in *ush2a^u507^* larvae. (**A**) and (**B**) Macular hair cell cross-sections from 6 days post fertilization (dpf) wt and *ush2a^u507^* larvae were immunostained with anti-acetylated tubulin (red), anti-usherin-N (green) and DAPI (blue). (**C**) and (**D**) Stereociliary hair bundles of the anterior macula in wt and *ush2a^u507^* zebrafish at 6 days post fertilization stained with Alexa Fluor 647 Phalloidin. Bar graph (**E**) shows the number of hair cell bundles per anterior macula in wt and *ush2a^u507^* larvae (mean ± SEM, *n* = 10). Bar graph (**F**) shows distance moved in response to acoustic stimuli of different frequencies (mean ± SEM, *n* ≥ 77 from three biological replicates per group). An unpaired *t*-test or two-way ANOVA were used to test for statistical significance. ^*^P < 0.05. Scale bars = 10 μm.

The localization of usherin was examined in the macular hair cells of 6 dpf zebrafish, using an N-terminal usherin antibody. In the wt hair cells, expression of usherin was concentrated at the region of the stereociliary hair bundles while specific expression was not detected in the *ush2a^u507^* larval macula (Fig. 7A and B). A similar expression pattern was observed in wt and mutant neuromasts (Supplementary Material, Fig. S6). The development of the anterior macula was examined in 6 dpf *ush2a^u507^* larvae using phalloidin staining (Fig. 7C and D). The hair bundles appeared slightly thinner in width and showed a mild but significant (*P* < 0.05) reduction in number in the mutant larvae compared to wt (61.90 ± 1.20 versus 67.6 ± 1.11, *n* = 10) (Fig. 7E).

Acoustic startle response testing was used to assess auditory function in 5 dpf larvae (Fig. 7F). When exposed to auditory stimuli of 100, 300, 600, 900 and 1200 Hz, *ush2a^u507^* fish moved a mean distance of 15.4 ± 0.86, 11.9 ± 0.80, 23.7 ± 1.48, 17.0 ± 0.95 and 17.2 ± 1.01 mm (*n* = 88), respectively, compared to 9.7 ± 0.65, 13.9 ± 1.00, 22.1 ± 1.41, 20.8 ± 1.33 and 21.5 ± 1.25 mm in wt (*n* = 77), respectively. The differences in response between wt and *ush2a^u507^* larvae were not found to be statistically significant.

Using scanning electron microscopy, the development of the neuromast hair cells was assessed by examining the ultrastructural morphology at 6 dpf (Supplementary Material, Fig. S6). In the wt hair cells, stereociliary hair bundles can be identified as tiers of stereocilia in staircase-like arrangements associated with a much longer kinocilium. In the *ush2a^u507^* neuromasts, stereocilia appear to be fewer and less well-organized within their bundles. Using FM1–43FX dye uptake to assess vesicle endocytosis in wt and *ush2a^u507^* neuromasts did not reveal significant differences (Supplementary Material, Fig. S6).

### Opsin mislocalization and autophagy defects in the *ush2a* mutant retina

**Figure 8:**
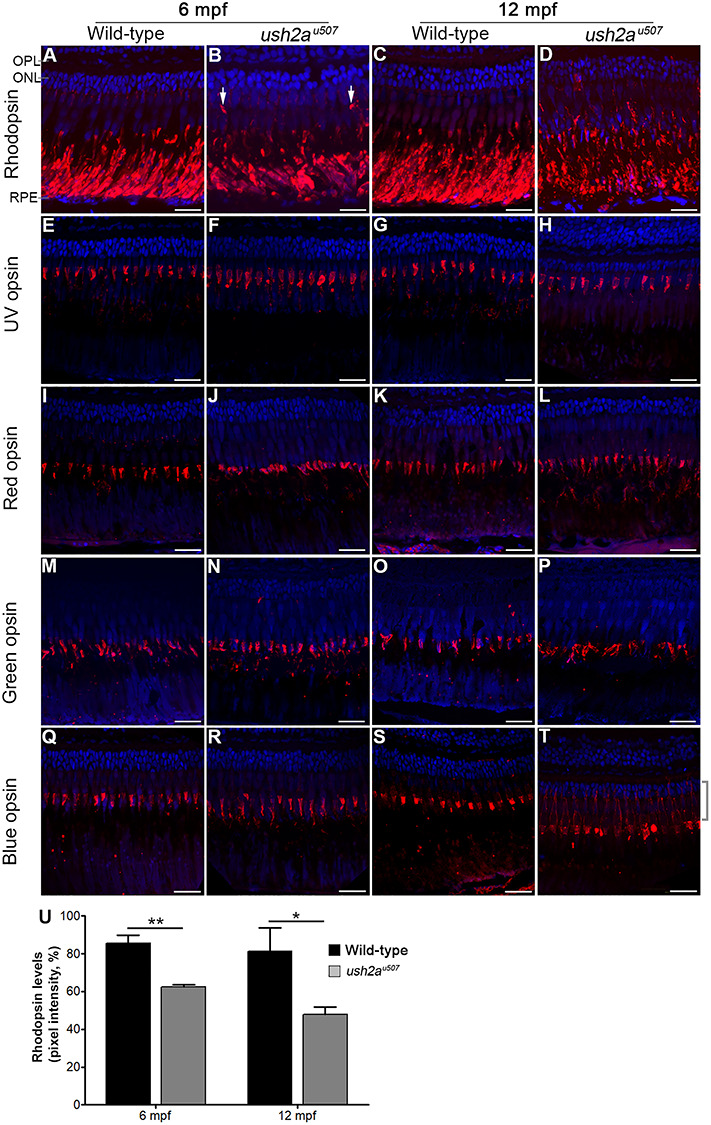
Rod and cone opsin localization in the *ush2a^u507^* retina. Rhodopsin (**A**–**D**) and UV (**E**–**H**), red (**I**–**L**), green (**M**–**P**) and blue (**Q**–**T**) cone opsins were detected in the wt and *ush2a^u507^* zebrafish retina at 6 months post fertilization (mpf), and 12 mpf by immunohistochemical analysis (red). In the 6 mpf *ush2a^u507^* retina, rhodopsin was partially mislocalized in some regions where it was detected in parts of the rod photoreceptors other than the outer segment (arrows in B). In the 12 mpf mutant retina, rhodopsin was more extensively mislocalized and expression in the rod outer segments was reduced compared to wt. Blue opsin was noted in the blue cone inner segments (highlighted with a bracket) in the *ush2a^u507^* retina at 12 mpf (T). All sections are counterstained with DAPI nuclei acid stain (blue). Bar chart (**U**) shows levels of rhodopsin detected in the rod outer segments at 6 and 12 mpf, measured using pixel intensity on confocal images (mean ± SEM. Three sections analyzed per fish, *n* = 3 per age). Unpaired *t*-tests were used to compare wt and *ush2a^u507^* rhodopsin levels at each age. ^*^P < 0.05, ^*^^*^P < 0.01. OPL, outer plexiform layer; ONL, outer nuclear layer; RPE, retinal pigment epithelium. Scale bar = 25 μm.

Rhodopsin was found to be mislocalized to the rod photoreceptor cell body in the *ush2a^u507^* mutant at 6 and 12 mpf, with significantly reduced protein expression levels by 26.8% (*P* < 0.01) and 35.9% (*P* < 0.05), respectively, compared to wt (*n* = 3) (Fig. 8A–D and U). Localization of the cone opsins was also assessed in the mutant retina (Fig. 8E–T). At 6 mpf, all cone opsins showed normal localization in both wt and *ush2a^u507^* retina. By 12 mpf, blue opsin showed accumulation in the blue cone inner segments in the *ush2a^u507^* retina. There did not appear to be notable differences in UV, red and green opsin localization between the wt and mutant retinas at the ages examined.

**Figure 9:**
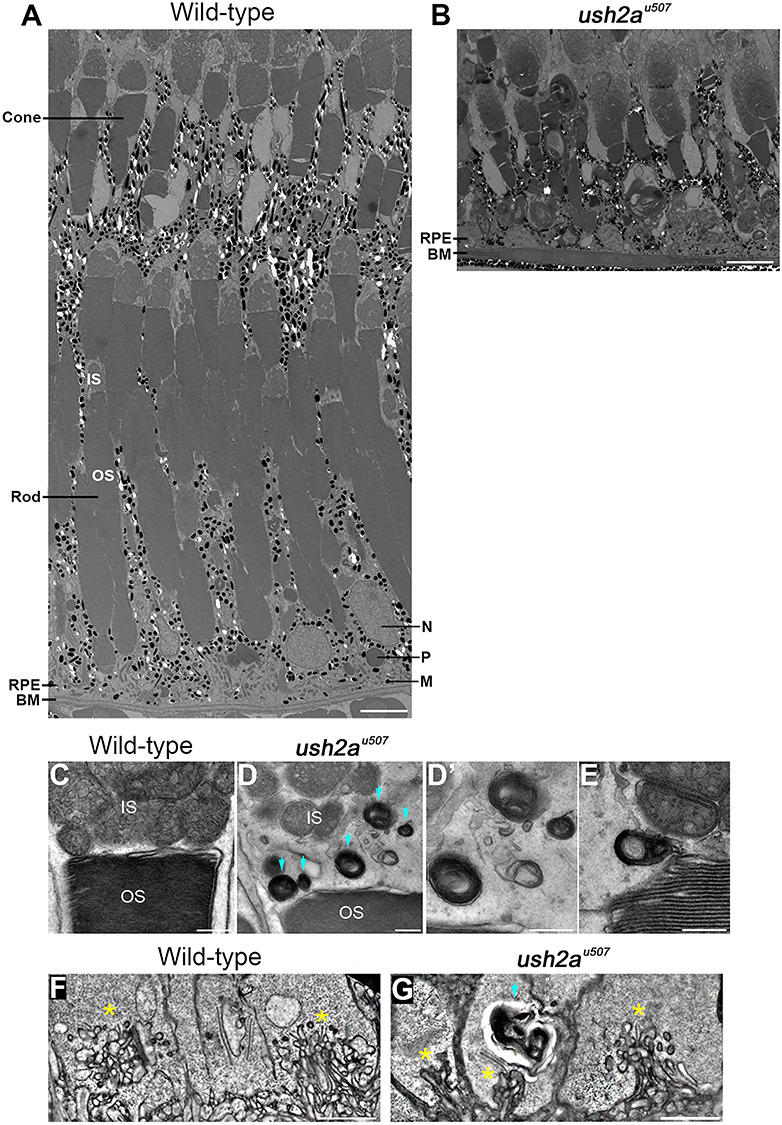
Retinal ultrastructure of *ush2a^u507^* zebrafish. Transmission electron microscopy was used to examine the wt and *ush2a^u507^* retinal ultrastructure at 6 months post fertilization (mpf). The wt retina showed tiers of morphologically distinct cone and rod photoreceptors with overlying retinal pigment epithelium (RPE) with long apical projections that interdigitated with the photoreceptors (**A**). Among mostly preserved tissue, regions of rod loss could be observed in the *ush2a^u507^* retina (**B**). Presumptive lysosomal structures (blue arrows) were noted at the inner and outer segment boundary (**D**, **E**) and at the synapses (**G**) of the *ush2a^u507^* photoreceptors at 6 mpf. Ribbon synapses are indicated by ^*^. These vesicles were not observed in wt photoreceptors (**C**, **F**). IS, inner segment; OS, outer segment; IS, inner segment; OS, outer segment; RPE, retinal pigment epithelium; BM, Bruch’s membrane; N, nucleus; P, phagosome; M, melanosome. Scale bars = 5 μm (A, B, F, G) and 500 nm (C–E).

Transmission electron microscopy was used to examine the retinal ultrastructure at 6 mpf, revealing patches of rod photoreceptor loss and cellular debris amongst mostly preserved areas of the *ush2a^u507^* retina (Fig. 9A and B). In addition, atypical vesicular structures, thought to be lysosomes, were noted around the photoreceptor inner and outer segment boundary and the ribbon synapses where the photoreceptors contact the bipolar and horizontal cells (Fig. 9C–G). These vesicles were not seen in age-matched wt retinas.

A comparison between the previously published *ush2a^rmc1^* mutant with the new *ush2a^u507^* line was undertaken to determine optimal outcome measures for future therapeutic testing. The ERG showed a stronger reduction of the b-wave amplitude in the *ush2a^rmc1^* mutant (10) and increased blue cone opsin mislocalization compared to strain-matched wt (*P* < 0.01) (Supplementary Material, Fig. S7). In *ush2a^u507^* larvae, no significant blue cone opsin mislocalization was observed as compared to strain-matched wt. Similarly, quantification of rhodopsin levels in the photoreceptor cell body revealed significantly increased rhodopsin mislocalization in the *ush2a^rmc1^* retina compared to controls (*P* < 0.05), but not in the retina of *ush2a^u507^* larvae.

**Figure 10:**
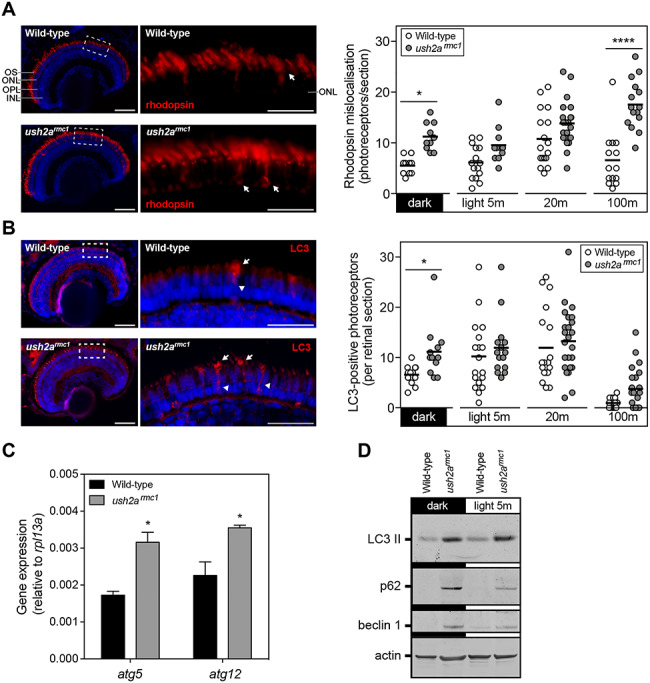
Rhodopsin mislocalization and elevated autophagy in *ush2a^rmc1^* larvae. (**A**) Rhodopsin (red) was predominantly detected in the outer segment of wt and *ush2a^rmc1^* larvae at 6 dpf. The inner segments occasionally revealed rhodopsin immunofluorescence, indicative of mislocalized rhodopsin transport vesicles (arrow). Representative images of dark-adapted larvae are shown for both genotypes. Quantification of the number of photoreceptors with distinct rhodopsin immunofluorescence in the inner segment revealed increased rhodopsin mislocalization in *ush2a^rmc1^* mutants. Two-way ANOVA revealed a significant interaction between the moment of sampling and the genotype of the larvae (*P* = 0.0019), a significant difference in rhodopsin mislocalization between wt and *ush2a^rmc1^* mutants (*P* < 0.0001) and difference in rhodopsin mislocalization depending on moment of sampling (*P* < 0.0001). Bonferroni’s post-test was used to reveal differences between groups (^*^P < 0.05; ^*^^*^^*^^*^P < 0.0001). (**B**) LC3 immunoreactivity, a marker for autophagosome vesicles, could be observed in the periciliary region (arrow) and inner segment (arrowhead) of a subset of photoreceptors. Representative images of dark-adapted larvae, are shown for both genotypes. Quantification of the number of photoreceptors with distinct LC3 immunofluorescence in the inner segment revealed increased presence of autophagosomes in *ush2a^rmc1^* mutants. Two-way ANOVA revealed no significant interaction between the moment of sampling and the genotype of the larvae, a significant difference in LC3-positive photoreceptors between wt and *ush2a^rmc1^* mutants (*P* = 0.0078) and difference in LC3-positive photoreceptors depending on moment of sampling (*P* < 0.0001). Bonferroni’s post-test was used to reveal differences between groups (^*^P < 0.05). (**C**) Quantitative RT-PCR analysis of autophagy associated genes *atg5* and *atg12* in wt and *ush2a^rmc1^* larvae. Two pools of 10 larvae were used per genotype (^*^P < 0.05, unpaired *t*-test). (**D**) Western blot analysis of pooled larvae, using antibodies directed at several markers of autophagy, revealed differences in expression of these markers between wt and *ush2a^rmc1^* mutants. Anti-actin was used as a loading control. OS, outer segments; ONL, outer nuclear layer; OPL, outer plexiform layer; INL, inner nuclear layer. Scale bars = 25 μm.

Zebrafish *ush2a^rmc1^* larvae (6 dpf) already showed significantly increased rhodopsin mislocalization (*P* = 0.016) prior to exposure of daily light levels (Fig. 10A); this was not found in wt controls. Twenty minutes after light onset, wt larvae showed rhodopsin mislocalization, which resolved by 100 min postlight onset; this rescue was not observed in *ush2a^rmc1^* mutants (*P* < 0.0001). Both sample timing related to light exposure and the *ush2a* genotype had a significant effect on the amount of rhodopsin mislocalization (*P* < 0.0001). Levels of rhodopsin mislocalization coincided with an increased number of autophagosomes in the inner segment of *ush2a^rmc1^* photoreceptors prior to light onset (*P* < 0.05, Fig. 10B), which was visualized by antibodies directed against autophagosome marker LC3. In wt larvae, the number of LC3-positive photoreceptors increased shortly after light onset and subsequently decreased to baseline levels by 100 min after light onset but remained slightly elevated in *ush2a^rmc1^* mutants (*P* = 0.11). Both the timing of sampling and the *ush2a* genotype were found to have a significant effect on the amount of LC3-positive photoreceptors (*P* < 0.0001 and *P* < 0.01, respectively). Expression of autophagy-associated genes *atg5* and *atg12* was significantly increased in light-adapted *ush2a^rmc1^* mutant larvae compared to wt controls (Fig. 10C). Western blot analysis revealed increased levels of lipidated autophagosomal membrane protein LC3 (LC3 II), p62/SQSTM and beclin 1 in *ush2a^rmc1^* larvae both before and after onset of light (Fig. 10D).

## Discussion

This is the first longitudinal study for Usher syndrome type II describing the retinal changes in a large *USH2A* patient cohort, and comparing it to *ush2a* zebrafish models to identify reliable therapeutic outcome measures for both preclinical and clinical studies. Our choice of EZ and hyperAF rings follows recent changes in Food and Drug Administration efficacy evaluation (23). This change represents recognition of the variability present in many functional markers such as visual acuity, microperimetry and visual fields. Evaluation of several clinical parameters showed that visual acuity did not change significantly over the 2–5 year follow-up period and retinal thickness was confounded by macular edema, which was not consistent with degeneration alone (Supplementary Material, Table S1). Neither of these metrics are suitable indicators of potential change in retinal function with disease progression, and thus, not suitable clinical endpoints. Unsurprisingly, there are statistically significant correlations between both the rate of change of the EZ length constriction and the hyperAF ring area with age. However, when evaluating the cohort for predictive value of these trends, it is clear that the strong statistical significance results from the large number of subjects. Considerable variability exists in the populations, exemplified by low *r* value describing the relationships. Therefore, trials aiming to predict future progression based on age-matched cohorts would require large numbers of participants in order to account for this variability. This is complicated by the fact that Usher syndrome is a slowly progressive, rare disease with a limited number of patients that can be recruited to any trial and the effect size of a novel treatment is difficult to predict. Previous gene therapy trials have aimed at slowing or halting disease progression (rather than attaining marked visual improvement). Although new trials provide promise that improvement may be possible (24), based on prevailing data both on acceptable endpoints and most probable results, EZ length constriction versus age has the strongest correlation and best repeatability However, taking this a step further to evaluate its use for a trial, this correlation only accounts for 44% of future variability. This means a trial designed in a 2:1 treated to untreated ratio, as a function of age, 612 total subjects (408:204) would be required to detect an 80% reduction in degeneration rate.

Furthermore, based on repeatability data, the trial would need to continue for 2 years in order to have detectable changes in a sufficient majority of patients. This detection of change and rate do scale with the size of baseline measurement as shown by the lack of taper in the repeatability data (Supplementary Material, Fig. S3), suggesting that all measurements have equal error and that absolute error does not change. Detection threshold is fairly constant across sizes of measurement as evaluated in actual values; however, if scaled as a percent of residual tissue, the larger the baseline measurement the smaller the possible error in detection. Additionally, degeneration is not linear with age, with patients experiencing quicker and slower degeneration rates at different stages of the disease. Therefore, trials designed based on younger patients degenerating rapidly may provide better possibility of success. We must await one of two results to confidently design a future clinical trial: (i) multifaceted longitudinal trials such as CRUSH and RUSH2A (NCT03146078), which will provide more data points allowing multilinear regression models to better inform proper endpoint and trial lengths and (ii) new artificial-intelligence-based methods (25,26) to design better predictors by using data on a particular solution to suggest an improvement in a specific outcome, thus altering the model away from simply reducing degeneration rate (20). It is likely that new data relating to both phenotype and each drug will be needed to accurately define future trials, due to differing targets and mechanisms of drug action, such as restoring function or slowing of progression. If gain of function is anticipated, trials could be relatively short (between 12 and 18 months) to arrive at an estimate of potential longevity. If slowing disease progression is predicted, then the data herein can be used to model projection for the treatment.

The audiograms overall remained stable throughout the life span of our relatively small patient cohort. This was consistent with previously published data for type II Usher syndrome (27), suggestive that audiometric outcomes are not useful outcome measures for trials. A previous study reported the presence of more severe hearing loss in the presence of two protein-truncating mutations, including homozygotes for the common c.2299delG mutation, than in those with non-truncating changes (28). However, this was not observed in our two patients with homozygous c.2299delG mutations, who displayed stable moderate hearing loss. In contrast, case 6 with c.2299delG, p.(Glu767Serfs21^*^) mutation and c.14911C>T, p.(Arg4971^*^) mutation had severe hearing loss from their first decade of life that progressed to profound in the second decade suggesting that additional factors may play a role in severity of hearing loss. Further investigation in larger patient cohorts is required to draw more consistent conclusions.

Potential defects in the auditory system in the newly developed *ush2a^u507^* mutant line were also investigated. Splayed stereociliary bundles have been observed in the hair cells of several Usher type 1 and 3 zebrafish models including *myo7aa* (29), *cdh23* (30), *ush1c* (31) and *clrn1* (32), but this morphology was relatively intact in the *ush2a^u507^* fish. At high frequencies, we observed a reduction in acoustic startle response in *ush2a^u507^* larvae, although not statistically significant; this is inconsistent with the findings in the TALENs-generated *ush2a^−/−^* mutant, where acoustic startle responses were significantly reduced at both ages examined (10 dpf and 6 mpf) (17). Measurements taken from older *ush2a^u507^* fish, when the inner ear is further developed, may be necessary to detect auditory defects. Auditory function was not investigated in *ush2a^rmc1^* and *ush2a^b1245^* mutants (10). Usherin is known to be transiently expressed in the stereociliary ankle links of the inner ear hair cells, supporting normal hair cell bundle development (14,15). Unless treatments can be given early enough (*in utero*), post-natal therapeutic strategies are unlikely to have a significant impact on hearing. Based on the data from the *USH2A* cohort, and the mild stereocilia phenotype of the zebrafish, it is difficult to determine good outcome metrics of therapeutic effect on auditory function at this moment. Additional tests such as otoacoustic emission and electrocochleography/auditory brainstem response in patients, or microphonic potential recordings in zebrafish, may provide a more detailed analysis of hair cell function (33,34).

Several *USH2A* animal models exist and have been characterized including the *Ush2a* null mouse (8) and zebrafish mutants (10,17). While these models have added to our understanding of the *USH2A* pathophysiology, the molecular pathways underlying retinal degeneration have not yet been elucidated. In *Ush2a* null mice, first evidence of retinal degeneration was observed at 20 months of age (8). In contrast, previously published zebrafish models (*ush2a^rmc1^*, *ush2a^b1245^* and *ush2a^hzu6^*) displayed early defects in retinal function (10,17). Unlike the *ush2a^rmc1^* and *ush2a^b1245^* mutants (10)*,* the *ush2a^u507^* zebrafish line presented here displayed a slowly progressive retinal degeneration, evidenced by an increased number of apoptotic photoreceptors and loss of ONL nuclei with age. Progressive degeneration was mentioned in the *ush2a^hzu6^* line (17), but their conclusions were only based on gross changes in retinal morphology. **ERG** recordings of *ush2a^u507^* larvae revealed a decrease in b-wave amplitude that is similar to that measured in *ush2a^hzu6^* larvae, but milder as compared to *ush2a^rmc1^* larvae.

Following the observed decrease in retinal function and retinal degeneration in *ush2a*^*u50*7^ zebrafish, the molecular defects resulting from loss of usherin function were further examined. Usherin was previously shown to be localized at the periciliary membrane of the photoreceptor cells, interacting with other USH2 proteins to form a complex that is hypothesized to contribute to the transfer of cargo, such as rhodopsin, from inner segment transport carriers to the ciliary transport system (9). This periciliary protein network was originally identified in frog photoreceptors, in an analogous area called the periciliary ridge complex (35), considered to be a docking site of vesicles transporting newly synthesized rhodopsin from the Golgi. Mislocalization of rhodopsin and blue cone opsin found in the *ush2a^u507^* retina contrasts with the findings from the *ush2a^hzu6^* mutant, which did not show photopigment mislocalization at any age (17), and *ush2a^rmc1^* and *ush2a^b1245^* were not previously investigated for transport defects (10). Defective rhodopsin trafficking is consistent with the proposed impaired function of the periciliary membrane complex. In *whirler* mouse (Usher syndrome type 2D) photoreceptors, rhodopsin was found to be mislocalized (36), as were both rhodopsin and blue cone opsin in *Myo7a* mutant mice (Usher syndrome type 1B) (36,37) and zebrafish (38). The proteins encoded by these mutant genes, whirlin and myosin VIIA, are among several Usher syndrome proteins found to interact with usherin at the mouse periciliary membrane complex (9,39). The high degree of similarity in opsin and rhodopsin mislocalization phenotypes between Usher models is unsurprising, as N-terminal protein-truncating mutations in *ush2a* were previously shown to result in loss of all members of the USH2 protein complex from the periciliary membrane complex (10). However, in contrast to the *ush2a^u507^* zebrafish retina, the *Ush2a* mouse was found to have normal rhodopsin labeling while red-green cone opsin staining showed mislocalization by p80 (40). The reason for this species discrepancy is unknown. Herein, further study of the *ush2a^rmc1^* retina has revealed mislocalization of rhodopsin and blue cone opsin in larval stages, suggesting a more severe phenotype possibly linked to genotype or the genetic background of the mutant line, but was not further investigated. It has been reported that rhodopsin preferentially enters the photoreceptor outer segment in the dark in wt retinae (41); however, the higher levels of mislocalization in *ush2a^rmc1^* mutants sampled in the dark with persistence following light exposure indicates that photoreceptors are not able to elicit a proper cellular response.

Previous data indicate that autophagy plays a crucial role in maintaining photoreceptor health by degrading the excess of phototransduction proteins, preventing their toxic accumulation in the inner segment, which would otherwise cause retinal degeneration (42–45). An increase of autophagosomes in the *ush2a^rmc1^* mutant retina prior to onset of light, combined with the increased expression of beclin1, which is essential for the packaging of autophagic proteins to a pre-autophagosomal structure (46), and the upregulation of the expression of the autophagy-associated genes *atg5* and *atg12*, suggests that autophagy is induced in response to rhodopsin mislocalization (47). However, the stabilization of p62 (Sequestosome-1/Sqstm), a selective autophagy receptor for ubiquitinated proteins that is normally degraded through autophagy along with its ligands, indicates that lysosomal degradation of autophagosomes could be hampered (47), and may explain the persistence of mislocalized rhodopsin following light exposure. The inability of *ush2a^rmc1^* photoreceptors to recover in the dark, and/or the constant consumption of excess energy to facility this recovery, is hypothesized to progressively induce pro-apoptotic signaling and retinal degeneration.

Examination of the retinal ultrastructure of the *ush2a^u507^* mutant revealed the presence of vesicular structures with the appearance of lysosomes around the photoreceptor inner and outer segment boundary and at the synaptic termini. While this could be interpreted as lysosomes functioning at the maximum of their capacity in the *ush2a*-deficient retina, further investigation is necessary. The observed changes in autophagy parameters could also occur when fusion of autophagosomes with lysosomes is defective. While many cellular stress pathways sequentially induce autophagy (at early time points to overcome cellular stress) and apoptosis (at late time points with high/prolonged cellular stress), the overall design of autophagy–apoptosis crosstalk is highly complex and primarily aims to reduce cell death (48).

Rhodopsin mislocalization and autophagy are useful functional outcome measures for monitoring response to treatment in preclinical zebrafish *ush2a* models. They also provide targets for small molecule drug development, but a careful balance is required to prevent activation of pro-apoptotic signaling pathways associated with high levels of cellular stress. The effect of light on rhodopsin transport and the subsequent intracellular responses indicate that a proper comparison between different mutant lines is only feasible when animals are raised under the exact same conditions. However, modulating the light intensity and regime provides the opportunity to increase the resolution of therapeutic experiments, for example in high-throughput drug discovery approaches.

In summary, due to more detailed phenotypic analysis of the *ush2a* mutant zebrafish retina, new insights into *USH2A*-related retinal disease have been gained, including photopigment mislocalization, elevated autophagy and subcellular trafficking defects, in keeping with other models of Usher syndrome. Therapeutic outcome measures would ideally include assessment of retinal function such as ERG recordings, analysis of rhodopsin and blue cone opsin mislocalization and autophagy. For patients, multimodal retinal imaging approaches may be the most sensitive measure of change, but prospective deep phenotyping studies will provide greater insights into the key functional clinical endpoints that will offer accurate efficacy metrics.

## Materials and Methods

### Subjects

Potential subjects were identified from the prospectively consented Moorfields Eye Hospital Inherited Eye Disease Database for structure/function of genetic diseases. This ongoing study is designed to facilitate discovery of new genes and aggregate data for longitudinal phenotype studies. Data for these studies are collected as part of standard of care and retrospectively analyzed. From this database, 56 *USH2A*-associated Usher Syndrome patients with molecularly confirmed biallelic *USH2A* mutations with at least three clinical visits close to a year between each visit (average 15 months between visits, range 9–51 months) were identified. A complete eye examination was performed in all subjects, including BCVA (converted to decimal units to facilitate comparison) and retinal imaging.

### Retinal imaging

Spectral domain optical coherence tomography (SD-OCT), (19 B-scans, 512 A-scans/B-scans; 97 B-scans, 1024 A-scans/B-scans) and fundus autofluorescence (FAF) imaging was performed on all patients using the Heidelberg Spectralis (Heidelberg Engineering, Heidelberg, Germany) with Automated Retinal Tracking. As this was a retrospective cohort, scan protocols were determined by the treating clinician at each visit and would receive one but not both scanning protocols. From these volumes the central SD-OCT B scan was identified by a trained observer as having the least residual inner retinal tissue and thickest ONL presence. For analysis, only scans with at least 500 μm of residual EZ present at baseline, but not so intact that it extended beyond the edges of the scan were included. FAF imaging was done using full power, blue light autofluorescence using the Heidelberg Spectralis (Heidelberg Engineering, Heidelberg, Germany) at 30 or 55° depending on which best visualized the residual FAF area.

All images were analyzed with Heidelberg Eye Explorer Region Finder Version 2.4.3.0 (Supplementary Material, Fig. S1) for measurements of retinal thickness and intact retinal area. The hyperautofluorescent (hyperAF) ring area as well as horizontal and vertical widths were manually segmented (Supplementary Material, Fig. S1D). All grading was done by two trained graders to ensure repeatability (Supplementary Material, Fig. S3).

### Audiology

Eleven patients had their pure tone audiograms undertaken by their local audiology service according to the British Society of Audiology recommended procedures (49). The pure tone average (PTA), which indicates the hearing level of the patient was calculated by averaging the hearing threshold in decibels hearing level (dB HL) at 500, 1000, 2000 and 4000 Hz frequencies. The level of hearing loss is categorized according to the PTA level as mild hearing loss 20–40 dB HL, moderate hearing loss 41–70, severe hearing loss 71–95 and profound hearing loss >95 dB HL. The configuration and symmetry of response between both ears was also analyzed.

### Zebrafish husbandry

Zebrafish [wt, AB-strain (wt) and *ush2a^u507^*] were bred and maintained at the UCL Institute of Ophthalmology animal facility. wt and *ush2a^u507^* zebrafish were generated by pair-wise matings of genotyped homozygous fish and raised at 28.5°C on a 14 h light/10 h dark cycle. Zebrafish [wt, TL-strain (wt) and *ush2a^rmc1^*] were generated and maintained as previously described (10). Heterozygous *ush2a* zebrafish were not examined in this study. Developing embryos were staged in days post fertilization (dpf) as previously described (50). For terminal experimentation, *z*ebrafish were anaesthetized in 0.2 mg/ml Tricaine (MS-222, Western Chemical Inc., Scottsdale, AZ, USA) and euthanized under schedule 1.

### CRISPR/Cas9 mutagenesis

To generate the *ush2a^u507^* mutant zebrafish, CRISPR targets were identified using an online software tool (https://zlab.bio/guide-design-resources) and the following 20 bp target guide sequence in *ush2a* exon 12 was chosen: 5′-AATATCTGGCTGCACGGTCC-3′. A cloning-free method similar to that reported in Gagnon *et al.* was used to synthesize the sgRNA (51). The Cas9 plasmid pT3Ts-nCas9n (Addgene #46757) was used as a template for Cas9 mRNA synthesis. A solution of 150 ng/μl Cas9 mRNA and 75 ng/μl sgRNA was co-injected into the cell of one-cell stage embryos from wt breedings, using a Picospritzer III microinjector (Intracel, Herts, UK). A dose volume of approximately 1 nl was used for injection.

To verify the mutagenesis activity of the sgRNA, genomic DNA was extracted from injected embryos at 24 hpf followed by PCR amplification of the region containing the target site. To extract DNA, de-chorionated embryos were incubated for 3 h at 50°C in DNA extraction buffer consisting of: 10 mm Tris (pH 8.2), 300 mm NaCl, 0.5% SDS, 200 μg/μl proteinase K and 10 mm EDTA. An *ush2a* DNA sequence of 652 bp containing the CRISPR target site was PCR amplified from the samples using MyTaq DNA polymerase (Bioline, London, UK) with the *ush2a* exon 12 primers, which spanned the target site. The sequences were as follows: 5′-GAGACCCATTCTGTTAAAGCACC-3′ and 5′-TCCCAGTTTGGCATTGCAGATC-3′. The cycle conditions used were: incubation at 95°C for 1 min for initial denaturation, followed by 35 cycles of 95°C for 15 s (denaturation), 61°C for 15 s (annealing) and 72°C for 10 s (extension). Samples were analyzed using Sanger sequencing. When mutations were detected, batches of injected embryos were raised to adulthood. F0 fish that transmitted germline mutations were outcrossed with wt fish. Heterozygous F1 progeny that were identified as possessing frameshift mutations were outcrossed with wt fish. F2 heterozygous fish carrying the same mutation were incrossed to produce F3 progeny. Homozygous fish and their wt siblings were used for subsequent breeding and phenotypic characterization.

### RNAscope assay

Enucleated adult zebrafish eyes (~6 months post fertilization, mpf), or whole 6 dpf larvae, were fixed in 4% PFA/PBS at 4°C overnight. After washing three times in PBS for 10 min, the eyes/larvae were incubated in 10, 20 and 30% sucrose/PBS at 4°C overnight each time. The samples were embedded in Tissue-Tek O.C.T embedding medium (VWR, Radnor, PA, USA) using dry ice and 12 μm sections were collected onto Superfrost^®^ PLUS slides (VWR) using a Leica CM1850 cryostat. The RNAscope assay was performed using the RNAscope Fluorescent Multiplex Detection v2 kit (ACD) as previously described (52). The *ush2a* target probe and *odc1* and *dapB* control probes were designed and provided by ACD.

### Retinal histology

For histological evaluation, enucleated eyes were fixed in 4% paraformaldehyde/PBS at 4°C overnight before processing and embedding using the JB-4™ embedding kit (Polysciences Inc., Warrington, PA, USA) with 7 μm thick sections as described (53). Sections were imaged on a Leica DMRB with Jenoptik D-07739 Optical System. Numbers of nuclei in the ONL were counted from histology sections that contained optic nerve, using ImageJ (National Institutes of Health, Bethesda, MD, USA).

### TUNEL assay

Fresh enucleated eyes were fixed with 4% PFA/PBS overnight at 4°C before incubation in 30% sucrose/PBS for 6 h at room temperature. The samples were mounted and frozen in TissueTek O.C.T using dry ice. About, 12 μm sections were cut using a Leica CM1850 cryostat and collected onto Superfrost^®^ PLUS slides. The TUNEL assay was performed as previously described (53) using the ApopTag^®^ Plus Fluorescein In Situ Apoptosis Detection Kit (Merck-Millipore, Burlington, MA, USA). Images were taken on a Zeiss LSM 700 AxioImage M.1 upright microscope. At least three retinal sections were assessed for the number of apoptotic photoreceptor nuclei per fish.

### Immunohistochemistry

Cryosections were prepared from adult enucleated eyes or whole larvae and immunostained as previously described (10,52). Primary antibodies used were 4D2 (1:200; Abcam, Cambridge, UK; ab98887), anti-UV opsin, anti-blue opsin, anti-red opsin (1:200; gifted by Professor David Hyde, University of Notre Dame), anti-usherin-C (1:500; Novus Biological, Littleton, CO, USA; 27 640 002), anti-usherin-N (1:200; gifted by Dr Jennifer Phillips, University of Oregon), anti-LC3 (1:200; Thermo Fisher Scientific, Waltham, MA, USA; PA1-16930), anti-centrin (1:500; Merck-Millipore; 04-1624) or anti-acetylated tubulin (1:200; Sigma-Aldrich, St Louis, MO, USA; T7451). Primary antibodies and appropriate secondary Alexa Fluor antibodies (1:500; Thermo Fisher Scientific) were diluted in antibody solution. The slides were imaged using a Leica LSM 700 confocal microscope or a Zeiss Axio Imager fluorescence microscope equipped with an Axiocam MRC5 camera. Rhodopsin levels were quantified using ImageJ. For this, all pictures were taken using the same settings after which the region of interest (adult retina: rod outer segments, larval retina: inner segment and ONL) was defined manually and assessed using the mean pixel intensity measurement.

### Western blot of autophagy markers

Pools of 50 larvae per group were sampled on ice in lysis buffer [2% Triton X-100 in Tris-Buffered Saline (TBS), pH 7.4, supplemented with protease inhibitor cocktail (Roche)], and homogenized using a pestle, and multiple passes through a small-gauge needle. Next, 13 μl of sample was mixed with 5 μl loading dye (LI-COR Biosciences, Lincoln, NE, USA) and 2 μl 1 m DTT, and incubated for 10 min at 70°C. Samples were separated on a 4–12% SDS-Page gel (Thermo Fisher Scientific) and transferred to a nitrocellulose membrane. For immunoblotting, the membrane was blocked with 5% blotting grade block buffer (Bio-Rad, Hercules, CA, USA) in PBS for 1 h at room temperature, after which primary antibodies were incubated in 0.5% blocking buffer overnight at 4°C. After washing three times with PBS supplemented with 0.2% Tween-20, the membrane was incubated with Alexa Fluor 680 or 800-conjugated secondary antibodies (Thermo Fisher Scientific, A21057, A21076 and LI-COR Biosciences, 926-32210) for 45 min at room temperature. After washing three times with PBS 0.2% Tween-20 and several rinses in PBS, membrane was scanned using the Odyssey CLx scanner (LI-COR Biosciences). The primary antibodies used were anti-LC3 (1:1000; Thermo Fisher Scientific; PA1-16930), anti-actin (1:2000; Abcam; ab3280), anti-P62 (1:1000; Cell Signaling Technology, Danvers, MA, USA; 5114T) and anti-BECN1 (1:1000; Santa Cruz, Dallas, TX, USA; sc-11 427).

### Electron microscopy

For transmission electron microscopy, enucleated zebrafish eyes were fixed in 2% paraformaldehyde-2% glutaraldehyde prior to incubation with 1% osmium tetroxide-1% potassium ferrocyanide. Following dehydration in an ethanol series and propylene oxide, the zebrafish were embedded in Epon 812 resin. Using a Leica EM UC7 ultramicrotome, 100 nm sections were cut, collected on formvar-coated copper grids (Electron Microscopy Sciences, Hatfield, PA, USA) and stained with lead citrate (Agar Scientific, Stansted, UK). Sections were examined on a Jeol 1010 TEM and imaged using a Gatan Orius SC1000B charge-coupled device camera.

For scanning electron microscopy, whole 6 dpf larvae were fixed and dehydrated as above. Samples were incubated twice in anhydrous methanol before hexamethyldisilazane (Sigma-Aldrich) incubation and overnight drying on watch glasses. Samples were mounted on stubs (Agar Scientific) and painted with conductive silver paint (Agar Scientific) and left to dry overnight before sputter-coating with gold/palladium using a Cressington Sputter Coater. Larval neuromasts were imaged on a Zeiss Sigma VP.

### Optokinetic response analysis

Adult zebrafish were lightly anaesthetized in 0.2 mg/ml tricaine and placed in a custom-made foam holder supported by dissection pins in a 55 mm petri dish. The dish was filled with tank water and the fish were allowed to regain consciousness, then placed into a custom-made optokinetic device consisting of a 12 cm acrylic drum, rpm adjustable rotating motor with laser tachometer and stereo microscope (Zeiss Stemi-2000C) c-mounted with a digital SLR camera (Nikon D5100). Each zebrafish was assessed with varying grating lengths at a consistent rpm speed (12 rpm) until the stripes could no longer be tracked by the zebrafish, as previously described (52).

### ERG recordings

ERG recordings were performed on isolated larval eyes (5 dpf) as previously described (10). Briefly, larvae were dark-adapted for a minimum of 30 min prior to measurement and subsequently handled under dim red illumination. The electrode was filled with E3 embryo medium (5 mm NaCl, 0.17 mm KCl, 0.33 mm CaCl, and 0.33 mm MgSO_4_) and positioned at the center of the cornea. A white light pulse (pE-4000, CoolLED) of 100 ms duration, with a light intensity of 600 lux was provided. Electronic signals were amplified 1000 times by an amplifier (EXT-02F, NPI electronic Instruments, Tamm, Germany) with a band pass between 0.1 and 700 Hz, digitized with Micro1401-3 (Cambridge Electronic Design Limited, Cambridge, UK) and displayed in Signal 6.03 (Cambridge Electronic Design Limited). The response to three consecutive pulses was averaged for each eye.

### Wholemount phalloidin staining of zebrafish larvae

Larval zebrafish at 6 dpf were fixed with 4% PFA/PBS overnight at 4°C. Zebrafish were washed in PBS for 10 min and incubated in PBS/1% Triton-X for 4 days to dissolve the otoliths. The larvae were washed three times for 10 min in PBS/1% Triton-X before incubating for 2 h at room temperature with Alexa Fluor 647 Phalloidin (Thermo Fisher Scientific) diluted 1:10 in 2% normal goat serum in PBS/0.5% Triton-X. The larvae were washed twice for 10 min in PBS/1% Triton-X before mounting in Prolong Gold Antifade Mountant on self-made multiwell microscope slides. Fluorescent hair bundles were visualized using Zeiss LSM 700 AxioImage M.1 upright confocal microscope.

### Acoustic startle response analysis

Experiments were performed using a vibration module and fast camera system (Viewpoint Life Sciences, Civrieux, France). The vibration module has a frequency range of 20–20 000 Hz, with variable intensities generated through a 40 W amplifier. The vibration time, duration and frequency were programmed using Audacity software (54). The vibration stimulus is synchronized with an infrared LED signal detected by the 1000 fps camera (Baumer, Frauenfeld, Switzerland) and automated software detects the C and S- start response of each fish in the well, as described in (55). A blinded study was used with wt and mutant groups at 5 dpf. Ten larvae were placed in each of 3 wells of a 6-well microtiter plate. A sequence of pure tone stimulations with frequencies of 600, 100, 900, 300 and 1200 Hz were generated. Each tone lasted 100 ms with a gap between each tone of 100 s, to avoid habituation. The response to each sound was recorded at 1000 fps and measurement of distance moved (mm) was performed by the Viewpoint software. The tone sequence was performed at the lower level able to produce a robust C-start response in wt fish.

### FM1–43 uptake

Long-term dye incorporation experiments were performed as described previously (56). Briefly, 6 dpf zebrafish larvae were pre-incubated for 10 min in BAPTA (Thermo Fisher Scientific) to disrupt the tip links and thus block FM1–43 entrance through the mechanotransduction channels, and then for 90 min with 3 μm of FM1–43FX (Thermo Fisher Scientific’) in the presence of low BAPTA concentrations for assessment of vesicle endocytosis. After several washes, animals were fixed with 4% PFA, counterstained with phalloidin and processed for fluorescence analyses. Fluorescence intensity was estimated using ImageJ as previously described (56).

### Autophagy gene expression analysis

Per genotype, RNA was extracted from 2 pools of 10 larvae (5 dpf) using Trizol reagent (Thermo Fisher Scientific) according to manufacturer’s instructions. RNA was further purified and DNase-treated using nucleospin RNA kit (Machery Nagel, Düren, Germany). Reverse transcription was performed with Superscript II (Thermo Fisher Scientific) in combination with random hexamers (Thermo Fisher Scientific) and RNaseOUT (Thermo Fisher Scientific). Quantitative RT-PCR was performed on a Biorad CFX-96 real-time PCR machine, using 5× diluted cDNA and GoTaq DNA polymerase (Promega, Madison, WI, USA). Primer sequences used are provided in Supplementary Material, Table S3.

### Statistics

Given the high degree of interocular symmetry in human data, the eye with the largest measurement baseline was used for all further analyses. To assess progression rate for each parameter, decline rates were binned by decade of age and averaged. Most measurements occurred at yearly increments; for those that were not, decline was assumed to be linear over the period of time between observations and therefore change was divided by time and rate of change/year was calculated. Pearson correlations were used to assess the relatedness between progression rates and parameters. All statistical analyses were done using JMP 13 (SAS Institute, Inc., Cary, NC, USA). Zebrafish data were shown as mean values ± SEM from *n* observations. The Shapiro–Wilk test was used to test for normal distribution. Student’s *t*-tests or Mann-Whitney *U* tests were used for single comparisons and two-way ANOVA followed by Bonferroni’s test was performed for multiple comparisons. *P* < 0.05 was accepted to indicate statistical significance (^*^). GraphPad Prism software was used for statistical analysis.

### Study approval

The patient study protocol adhered to the tenets of the Declaration of Helsinki and received approval from the NRES Ethics Committee (REC12/LO/0141). Written informed consent was obtained from all participating individuals prior to inclusion in study.

Zebrafish were maintained according to institutional regulations for the care and use of laboratory animals under the UK Animals Scientific Procedures Act (UCL Institute of Ophthalmology animal facility) and Dutch experiments on animals act (Radboud University Medical Center), UCL Animal Welfare and Ethical Review Body (License no. PPL PC916FDE7) and Animal Experiment Committee of the Radboud University Nijmegen (License no. RU-DEC, 2012-301). All approved standard protocols followed the guidelines of the ARVO Statement for the Use of Animals in Ophthalmic and Vision Research Ethics.

## Abbreviations

ADGRV1, adhesion G protein-coupled receptor V1, ANOVA, analysis of variance, BCVA, best-corrected visual acuities, bp, base pairs, cpd, cycles per degree, CRISPR/Cas9, clustered regularly interspaced short palindromic repeats/CRISPR-associated protein 9, DAPI, 4′,6-diamidino-2-phenylindole, dpf, days post fertilization, ERG, electroretinogram, EZ, ellipsoid zone, FDA, Food and Drug Administration, GCL, ganglion cell layer, hyperAF, hyperautofluorescent, Hz, hertz, INL, inner nuclear layer, IPL, inner plexiform layer, IS/OS, inner/outer segments, mpf, months post fertilization, OCT, optical coherence tomography, ON, optic nerve, ONL, outer nuclear layer, OPL, outer plexiform layer, PAM, protospacer adjacent motif, PTA, pure tone average, RP, retinitis pigmentosa, RPE, retinal pigment epithelium, rpm, revolutions per minute, RT-PCR, reverse transcription polymerase chain reaction, SEM, standard error of the mean, TUNEL, terminal deoxynucleotidyl transferase dUTP nick end labeling, USH2, Usher syndrome type 2, wt, wild-type

## Supplementary Material

Toms_et_al_Supplementary_Material_ddaa004
