# Supplementary material for: Clinical and preclinical therapeutic outcome metrics for USH2A-related disease
Source: Hum Mol Genet. 2020 Jan 30;29(11):1882–99. doi: 10.1093/hmg/ddaa004 (PMC7372554; doi:10.1093/hmg/ddaa004)
Supplement: Toms_et_al_Supplementary_Material_ddaa004 [file toms_et_al_supplementary_material_ddaa004.pdf]

<u>Supplementary material</u>

Clinical and preclinical therapeutic outcome metrics for *USH2A*-related disease

Maria Toms, Adam M Dubis, Erik de Vrieze, Dhani Tracey-White, Andreas Mitsios, Matthew Hayes, Sanne Broekman, Sarah Baxendale, Nattawan Utoomprurkporn, Doris Bamiou, Maria Bitner-Glindzicz, Andrew R Webster, Erwin Van Wijk, Mariya Moosajee

Supplementary Table S1. Patient Demographics and Imaging Data

1 = Age (years); 1a = Duration between visits (years); 2 = HyperAF area (mm^2); 3 = HyperAF horizontal diameter (µm); 4 = HyperAF Vertical Diameter (µm); 5 = EZ Length (µm); 6 = Retinal Thickness ILM to EZ (µm); 7 = Retinal Thickness EZ to RPE (µm).

					Visit 1							Visit 2								Visit 3							
Subject Data			Visual Acuity (Decimal)			Autofluorescence Measurements			OCT Measurements					Autofluorescence Measurements			OCT Measurements					Autofluorescence Measurements			OCT Measurements		
	Allele 1	Allele 2	Baseline	Follow Up	1	2	3	4	5	6	7	1	1a	2	3	4	5	6	7	1	1a	2	3	4	5	6	7
1	c.5836C>T	c.2299delG	0.3	0.3	44	3.60	2391	1939	1548	184	61	45	0.92	3.33	2126	1845	1476	180	57	46	0.92	3.10	2103	1834	1400	176	88
2	c.4821G>A	c.1859G>T	1	0.7	52	5.76	3064	2522	2316	180	73	53	0.92	5.59	3035	2404	2081	176	71	55	2.08	5.24	2933	2370	2049	176	110
3	c.2610C>A	c.2610C>A	0.25	0.5	25	50.09	8048	7435	5921	201	71	27	1.75	39.45	8045	6690	5757	200	67	28	1.42	31.34	6544	5586	5651	201	65
4	c.13130C>A	c.1256G>T	0.7	0.7	34	4.66	2842	2183	2567	275	71	35	1.08	4.26	2717	2023	2384	212	68	36	1.25	3.99	2501	1936	2309	223	65
5	c.1876C>T	c.2299delG	0.7	0.7	34	2.17	1855	1458	994	161	63	36	1.25	1.80	1751	1292	823	145	59	37	1.42	1.28	1330	1108	716	137	53
6	c.14911C>T	c.2299delG	0.5	0.7	36	3.64	2501	1751	3088	155	76	37	0.92	3.31	2001	1641	2788	148	73	38	0.92	2.01	1751	1208	2383	160	204
7	c.6967C>T	c.7883dupC	1	1	37	7.06	3622	2561	3510	216	69	38	1.00	6.79	3592	2455	3412	216	67	39	0.92	6.33	3495	2439	2862	392	188
8	c.11864G>A	c.13335_13347delinsCTTG	0.7	0.7	32	6.19	3127	2459	2813	196	65	33	0.92	5.43	2863	2251	2636	209	63	34	1.17	4.85	2813	2068	2356	175	65
9	c.11700C>A	c.11700C>A	0.3	0.2	39	3.95	2510	2176	1997	563	55	40	0.67	3.91	2411	1925	1863	539	52	44	4.25	3.10	2126	1668	1603	167	63
10	c.9976C>T	c.9976C>T	0.5	0.5	50	24.16	6585	5156	1689	306	57	51	0.92	21.17	6374	4814	1600	293	56	53	1.58	18.08	5502	4189	1557	220	80
11	c.100C>T	c.926C>T	0.5	0.5	53	0.97	1250	990	955	112	57	54	1.17	0.91	1046	910	827	109	52	55	1.17	0.90	1023	841	591	98	67
12	c.7789A>T	c.7789A>T	0.5	0.25	61	1.64	1671	1330	1331	131	64	64	3.00	1.42	1500	1125	965	122	60	65	0.92	1.33	1396	1000	945	117	55
13	c.3841A>T	c.3840G>C	0.5	0.3	64	3.90	2313	1934	1710	161	60	65	0.92	3.60	2228	1876	1521	169	59	66	1.17	3.44	2149	1808	1406	166	80
14	c.4474G>T	c.2276G>T	0.7	0.7	26	6.00	2741	2887	4436	133	65	27	1.58	4.31	2449	2293	4137	129	64	29	1.50	3.17	2168	2032	3506	127	60
15	c.2023C>T	c.2299delG	0.5	0.3	52	1.79	1705	1271	1092	190	56	55	3.42	1.16	1390	961	903	172	47	57	2.00	0.98	1276	888	962	152	44
16	c.8079G>A	c.12575G>A	0.07	0.05	46	0.18	485	455	681	130	28	47	1.00	0.07	286	240	578	133	24	48	0.92	0.06	276	224	428	151	62
17	c.11065C>T	c.7645_7661del	1	1.2	16	24.95	5281	5654	3982	151	67	19	3.00	7.02	3432	2817	3435	139	56	21	1.75	4.34	2863	2022	2926	135	161
18	c.8981G>A	c.13274C>T	1.2	0.7	49	6.26	2957	2778	4803	173	63	50	1.00	5.33	2768	2617	4548	167	60	52	1.92	4.39	2536	2366	4148	160	170
19	c.3841A>T	c.3840G>C	0.01	0.03	56	1.71	1511	1273				62	5.50	0.22	656	417				63	1.00	0.13	417	354			
20	c.11156G>T	c.14285A>G	0.1	0.1	41	5.10	2897	2114	2444	125	60	43	1.58	4.73	2644	2098	2052	104	56	46	2.75	1.04	1340	911	1737	96	55
21	c.10073G>A	c.920_923dupGCCA	0.7	0.7	32	3.86	2433	1984	1623	177	58	33	1.00	3.60	2318	1880	1466	169	54	34	1.42	3.40	2232	1752	1409	167	52

22	c.5603T>G	c.10996T>G	1.2	1.2	47	27.70	5713	6203	4341	149	65	48	0.92	20.78	5047	5978	3969	143	63	50	1.42	12.20	4109	4728	3713	155	188
23	c.10073G>A	c.11156G>A	0.7	0.45	57	1.69	1625	1272	903	106	52	58	1.33	1.59	1562	1213	839	95	51	59	0.92	1.42	1456	1203	737	91	163
24	c.10561T>C	c.7595-2144A>G	0.7	0.3	25	9.39	4608	2958	3093	165	53	27	1.83	6.90	3598	2462	2744	154	50	28	0.92	4.35	2846	1958	2691	134	78
25	c.2802T>G	c.2802T>G	1	1	43	6.49	3537	2390	2596	172	64	44	0.92	6.32	3312	2209	2373	171	63	46	1.92	6.06	3113	2149	2196	171	57
26	c.1036A>C	c.13316C>T	0.7	0.7	47	5.59	2689	2423	1975	185	47	49	1.92	4.27	2449	2126	1846	193	45	52	2.50	3.11	2074	1855	1876	176	127
27	c.5555A>G	c.13331C>T	1	1	37	38.26	7520	6368	5112	149	66	38	0.92	37.79	7440	6260	4805	151	63	39	0.92	32.96	7015	5992	4458	180	57
28	c.13274C>T	c.11875_11876delCA	1	1	39	9.39	4102	2734	2400	179	59	40	0.92	6.75	3597	2425	2212	174	54	41	1.17	5.09	3143	2089	2028	169	51
29	c.6854A>G	c.6653T>C	0.7	0.7	36	7.71	3274	2936	2569	154	61	38	1.92	6.48	3062	2659	2266	150	59	39	1.25	6.28	3057	2643	2140	135	182
30	c.2276G>T	c.4714C>T	1	0.7	35	4.87	2631	2314	1499	173	64	36	0.92	4.34	2619	2065	1416	185	61	39	2.25	3.91	2549	1971	1338	169	60
31	c.1256G>T	c.13750dupA	0.3	0.3	31	6.17	3165	2546	2022	140	53	32	0.92	5.92	3124	2308	1679	140	51	33	0.92	5.66	3041	2260	1487	145	150
32	c.13316C>T	c.2276G>T	0.7	0.7	41	3.33	2231	1912	1497	114	62	42	0.92	3.00	2167	1762	1365	128	59	45	2.75	2.05	1818	1393	1243	95	56
33	c.2276G>T	c.6470delG	0.5	0.5	25	8.03	3663	2897	2463	118	63	26	0.92	6.45	3298	2630	2165	113	62	27	1.42	5.29	3110	2385	2044	109	59
34	c.2276G>T	c.14426C>T	0.25	0.3	52	5.52	3079	2307	2050	262	53	53	1.25	4.82	3026	2070	1796	301	53	54	1.17	4.53	2707	2047	1695	210	50
35	c.4714C>T	c.7595-2144A>G	0.7	0.7	37	8.26	3788	2743	2423	120	52	38	1.42	6.83	3543	2533	2321	105	51	39	0.92	6.11	3093	2504	2110	100	163
36	c.10073G>A	c.11549-1G>A	0.7	0.7	43	23.30	6196	4578	5172	130	69	44	0.92	21.73	5895	4463	5050	137	67	47	2.67	17.46	5259	4205	4314	124	57
37	c.2299delG	c.2299delG	0.7	0.5	39	1.47	1610	1063	1162	128	67	41	1.92	1.38	1421	968	1003	128	65	43	1.42	1.22	1372	948	939	123	57
38	c.2299delG	c.2299delG	0.7	0.7	28	4.71	3102	1995	2443	190	64	30	1.50	3.80	2627	1752	2264	180	60	31	1.00	3.71	2533	1751	2017	177	88
39	c.2299delG	c.4714C>T	1	1	15	25.80	6406	5264	3908	200	64	16	1.00	24.84	6324	5131	3649	191	61	18	1.50	17.20	5711	3907	2711	218	180
40	c.2299delG	c.7595-2144A>G	0.3	0.2	37	5.82	3230	2229	2159	167	54	38	0.83	5.62	3206	2119	1942	140	52	39	1.58	5.47	3175	2110	1674	128	48
41	c.2299delG	c.4714C>T	0.5	0.3	41	5.03	2700	2210	1926	148	56	43	1.67	4.48	2667	2149	1761	158	54	45	1.92	3.82	2386	1873	1669	146	51
42	c.1679delC	c.6795_6797delATA	0.2	0.2	44	2.91	2019	1825	1585	199	53	46	1.92	2.84	2003	1816	1361	172	52	47	0.92	2.78	1984	1680	1119	159	145
43	c.2299delG	c.1606T>A	0.3	0.25	39	4.47	2437	2163	1027	100	50	40	1.00	3.47	2337	1963	886	94	48	42	1.42	2.99	2258	1625	908	85	71
44	c.2299delG	c.4510dup	1.2	1.2	36	6.66	3569	2632	2395	146	59	38	1.92	6.48	3362	2546	2212	154	53	39	0.92	6.42	3317	2471	2145	147	153
45	c.13374delA	c.2276G>T	1.2	1.2	43	11.08	4168	3504	3490	143	65	44	1.00	9.68	3862	3278	3040	141	62	45	1.25	9.04	3755	3130	2919	147	167
46	c.2299delG	c.5614_5620delGCTGTCG	0.7	0.7	33	5.61	3194	2302	2186	93	51	35	1.92	5.19	3181	2034	1998	88	47	36	0.92	5.00	2905	2024	1871	86	47
47	c.2299delG	c.1256G>T	0.02	0.02	41	6.90	3436	2472	2077	123	64	42	1.00	6.08	3308	2342	1947	108	63	46	3.17	4.99	3010	2026	1823	120	91
48	c.11694delC	c.3158-6A>G	1	0.7	32	11.67	4657	3979	3301	159	65	33	1.08	10.45	4303	3171	2924	156	61	35	2.00	9.02	4055	2841	2410	155	90
49	c.12819A>T	c.1055T>A	0.03	0.03	61	1.51	1485	1406	3036	131	75	62	1.00	1.47	1357	1396	2878	132	70	63	0.92	1.09	1263	1284	2854	136	100
50	c.9860_9873del ATGATGGCC ATGGC	c.6730G>A	0.5	0.5	28	6.63	3511	2389	2918	159	67	29	0.83	5.98	3224	2365	2651	141	65	32	2.83	5.87	3090	2356	2619	139	208
51	c.2299delG	c.4714C>T	0.3	0.3	25	4.63	2789	2052	2174	129	70	26	1.08	4.09	2611	1856	2097	128	64	27	1.25	3.21	2118	1821	1781	119	62
52	c.2081G>A	c.4714C>T	0.7	0.5	27	36.76	6885	6738	5583	188	69	29	1.92	30.22	6243	5962	5419	184	67	30	1.00	24.54	5611	5281	5024	180	96
53	c.4714C>T	c.14426C>T	0.7	0.7	55	3.71	2550	1915	1648	91	60	57	1.92	3.36	2294	1772	1506	102	54	58	0.92	2.33	2067	1448	1472	96	53
54	c.2299delG	c.2299delG	0.7	0.3	27	1.14	1333	1030	971	184	50	28	1.08	1.11	1289	1021	890	173	49	29	0.50	0.97	1273	898	798	165	48
55	c.2299delG	c.4714C>T	0.2	0.2	35	8.28	3599	2722	2748	243	41	36	1.00	7.28	3424	2634	2364	180	40	37	0.92	6.23	3206	2405	1927	161	60
56	c.895delC	c.2994A>T	0.2	0.1	20	63.13	8438	8584	4820	275	69	21	1.08	35.70	7092	6624	3383	355	67	24	3.17	25.21	6587	5340	3210	392	67

Supplementary Table S2. Age, genotype and audiogram measurements in *USH2A* patients. PTA, pure tone audiometry.
*patients not included in OCT analysis

Subject	Age of last audiogram	PTA (Right)	PTA (Left)	Severity (Right/Left)	Configuration	Symmetrical	Mutation cDNA	Mutation protein	Mutation cDNA	Mutation protein
2	55 yr	73.75dBHL	78.75dBHL	Severe/Severe	High frequency loss	yes	c.1859G>T	p.Cys620Phe	c.4821G>A	p.Trp1607*
5	38 yr	77.5dBHL	67.5dBHL	Severe/Moderate	High frequency loss	yes	c.2299delG	p.Glu767Serfs21*	c.1876C>T	p.Arg626*
6	38 yr	111.25dBHL	112.5dBHL	Profound/Profound	High frequency loss	yes	c.2299delG	p.Glu767Serfs21*	c.14911C>T	p.Arg4971*
15	49 yr	77.5dBHL	83.75dBHL	Severe/Severe	High frequency loss	yes	c.2299delG	p.Glu767Serfs21*	c.2023C>T	p.Gln675*
17	15 yr	65dBHL	61.26dBHL	Moderate/Moderate	High frequency loss	yes	c.7645_7661del		c.11065C>T	p.Trp3521Arg
37	43yr	61.25dBHL	55dBHL	Moderate/Moderate	High frequency loss	yes	c.2299delG	p.Glu767Serfs21*	c.2299delG	p.Glu767Serfs21*
38	31 yr	63.75dBHL	66.25dBHL	Moderate/Moderate	High frequency loss	yes	c.2299delG	p.Glu767Serfs21*	c.2299delG	p.Glu767Serfs21*
40	40 yr	80dBHL	93.75dBHL	Severe/Severe	High frequency loss	yes	c.2299delG	p.Glu767Serfs21*	c.7595-2144A>G	p.Lys2532Thrfs65*
57*	31 yr	45dBHL	40dBHL	Moderate/Moderate	High frequency loss	yes	c.2299delG	p.Glu767Serfs21*	c.820C>G	p.Arg274Gly
58*	37 yr	55dBHL	53.75dBHL	Moderate/Moderate	High frequency loss	yes	c.2299delG	p.Glu767Serfs21*	c.7595-2144A>G	p.Lys2532Thrfs65*
59*	20 yr	61.25dBHL	60dBHL	Moderate/Moderate	Flat	yes	c.920_923dupGCCA	p.His380GlnInfs16*	c.3518C>A	p.Ser1173*

Supplementary Table S3. Quantitative RT-PCR primer sequences

Gene	Forward primer (5' – 3')	Reverse primer (5' – 3')
atg5	TGAGCTCAACTGTAATGCTTCCT	GTCTTCAGCTACCAATCTTTGGA
atg12	GACGATACAGTCACTCGCCC	CGAAACACTCAAAAAGCACACC
rpl13a	TCTGGAGGACTGTAAGAGGTATGC	AGACGCACAATCTTGAGAGCAG

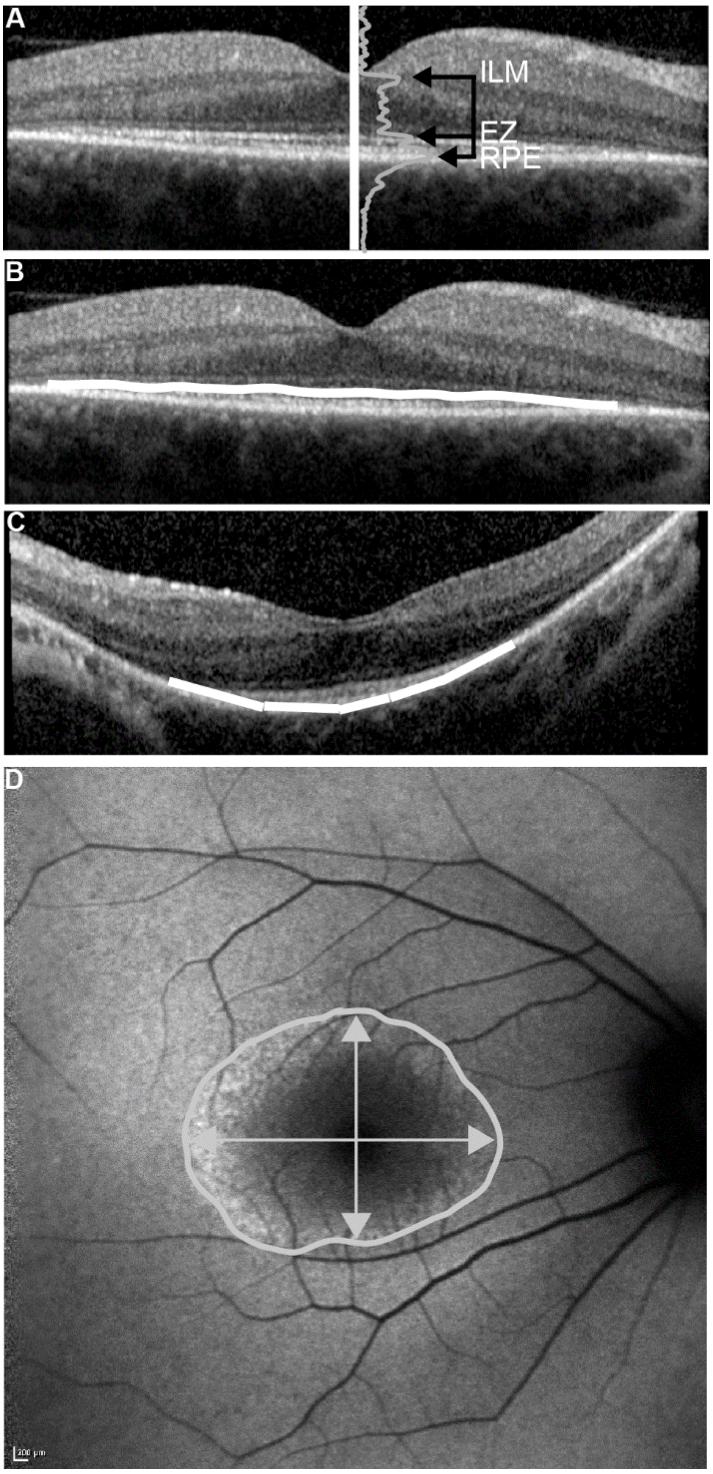

Supplementary Figure S1. Retinal image quantification.

(A) Measurement of central retinal thickness. Retinal thickness measurements were done at the foveal centre (yellow line). The definition of retinal lamina is illustrated by the peaks in intensity along the longitudinal reflectivity profile (white line) between internal limiting membrane (ILM) and ellipsoid zone (EZ) and retinal pigment epithelium (RPE). (B, C) Measurement of EZ length along the foveal scan for flat retinas (B) and curved retina (C). For curved retinal images, the summation of discrete measurements along the curve was used as EZ length. (D) Manual delineation of hyperautofluorescence ring (grey circle). Horizontal and vertical dimensions were measured as illustrated by the grey, arrow pointed lines.

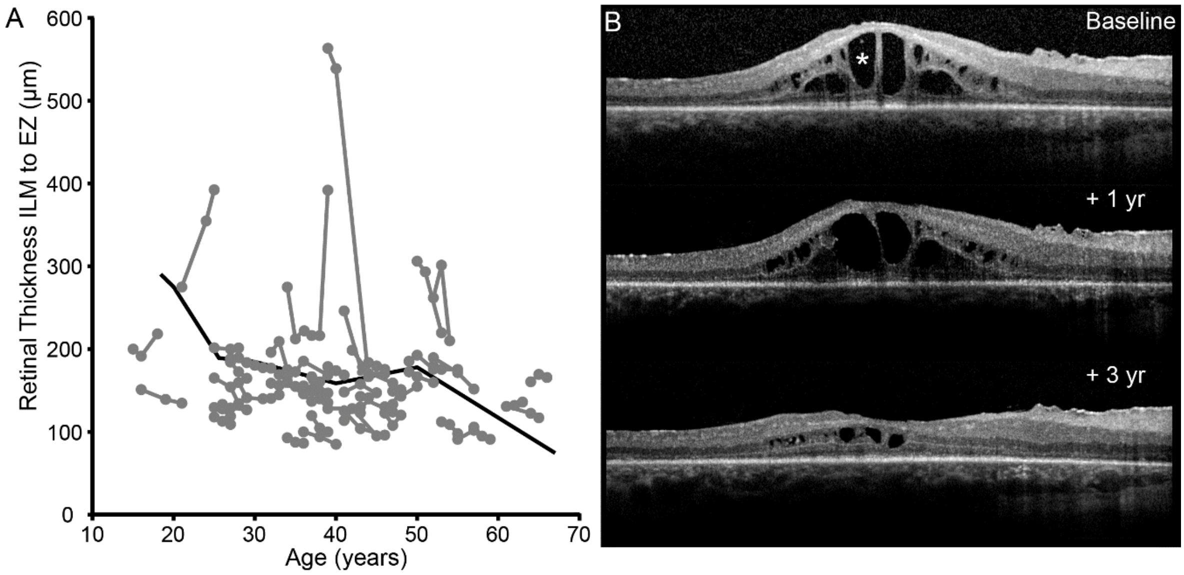

Supplementary Figure S2. Retinal thickness as a function of age and presence of cystoid macular edema.

(A) Changes in retinal thickness (internal limiting membrane (ILM) to ellipsoid zone (EZ) for all patients. Several patients show increase of retinal thickness or retinal thicknesses >350um, not consistent with normal retinas or degeneration. These variations are due to changes in macular edema (dark areas within the retina, highlighted with * on top image) status (B).

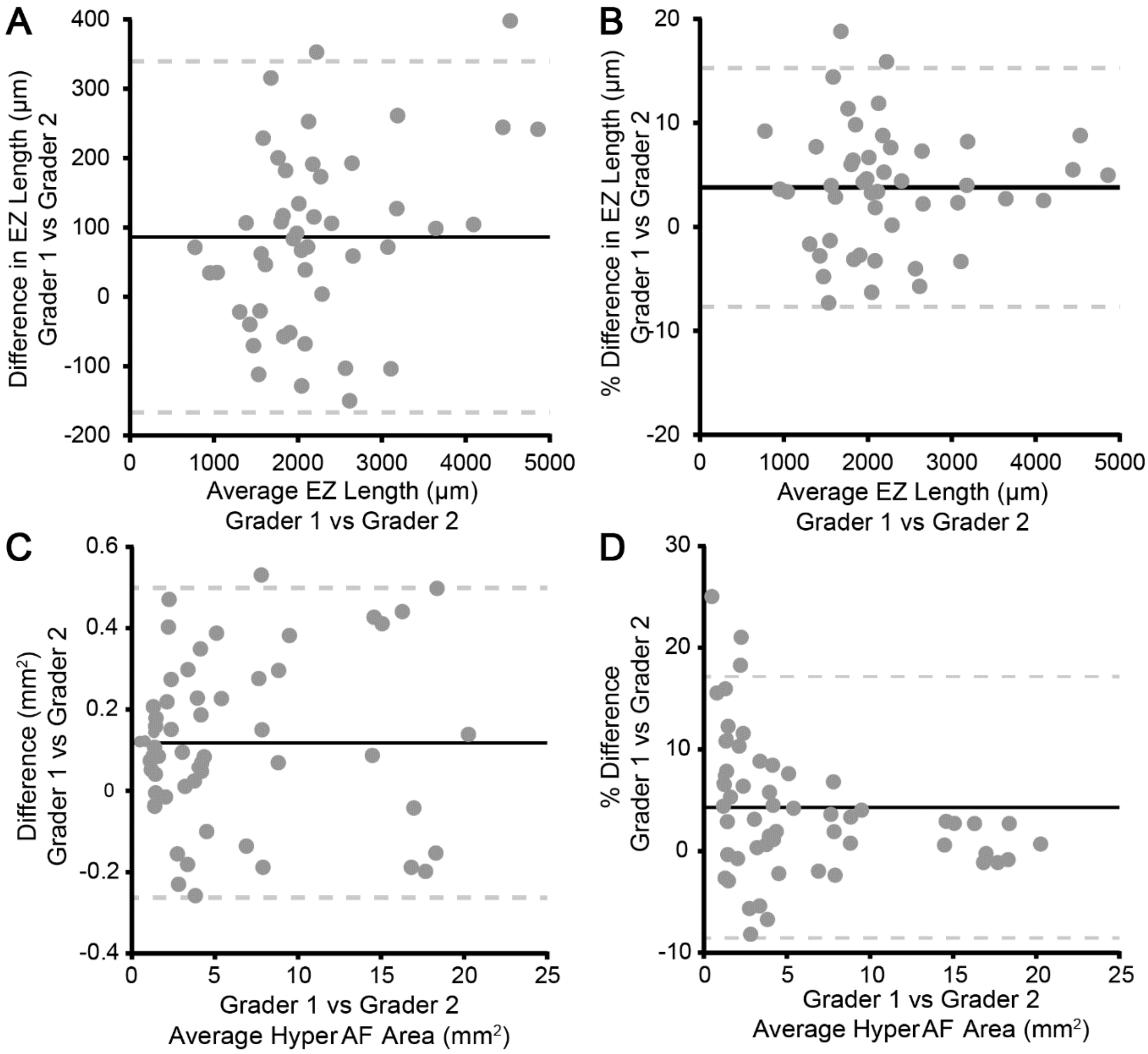

Supplementary Figure S3. OCT repeatability.

Bland-Altman plots show measurement differences between graders represented as both absolute value (A, C) and percent of total area (B, D). Differences between measurements for ellipsoid zone (EZ) length (A) appear to scale well across lengths, however when transformed to look at difference between graders and a function of total area (B) there is a clear indication that smaller EZ lengths are prone to greater percent difference error than larger lengths. This relationship is even stronger for hyperautofluorescence (hyperAF) area (C) and the percent difference (D).

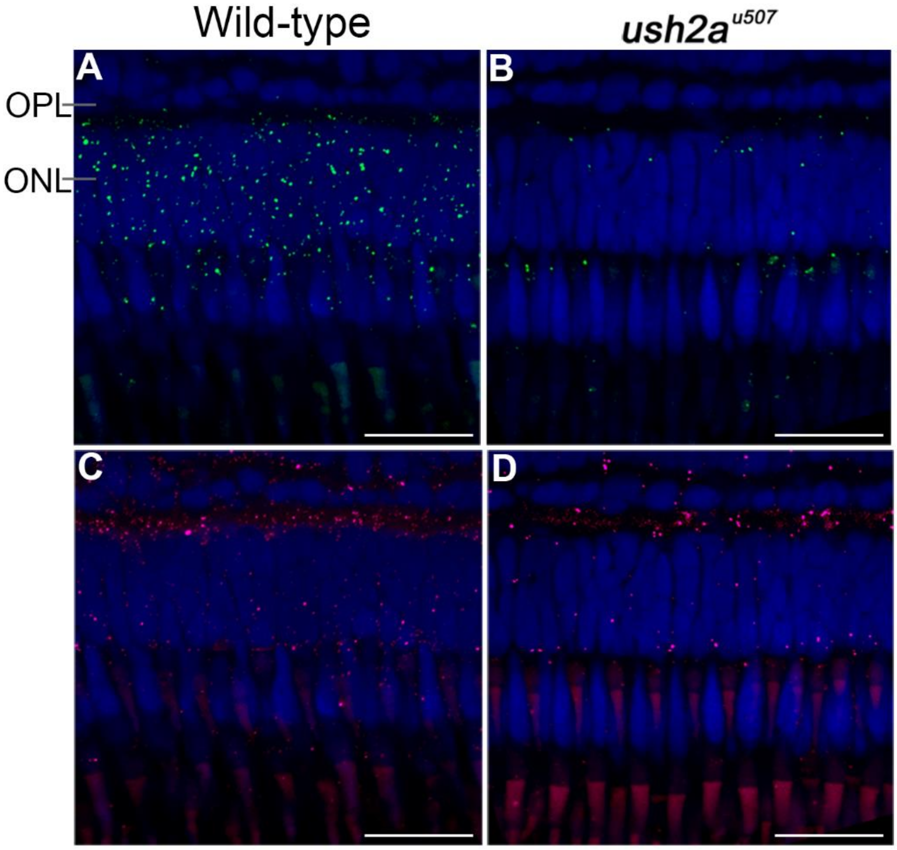

Supplementary Figure S4. ***ush2a*** **mRNA expression in the** ***ush2a***^{u507} **retina.**

Using the RNAscope assay, reduced *ush2a* mRNA (green) was detected in the adult mutant retina compared to wild-type (A, B). The positive control *odc1* (magenta) showed similar expression levels in both (C, D). DAPI nucleic acid stain was used as a counterstain (blue). PAM, protospacer adjacent motif. OPL, outer plexiform layer; ONL, outer nuclear layer. Scale bar = 25 µm.

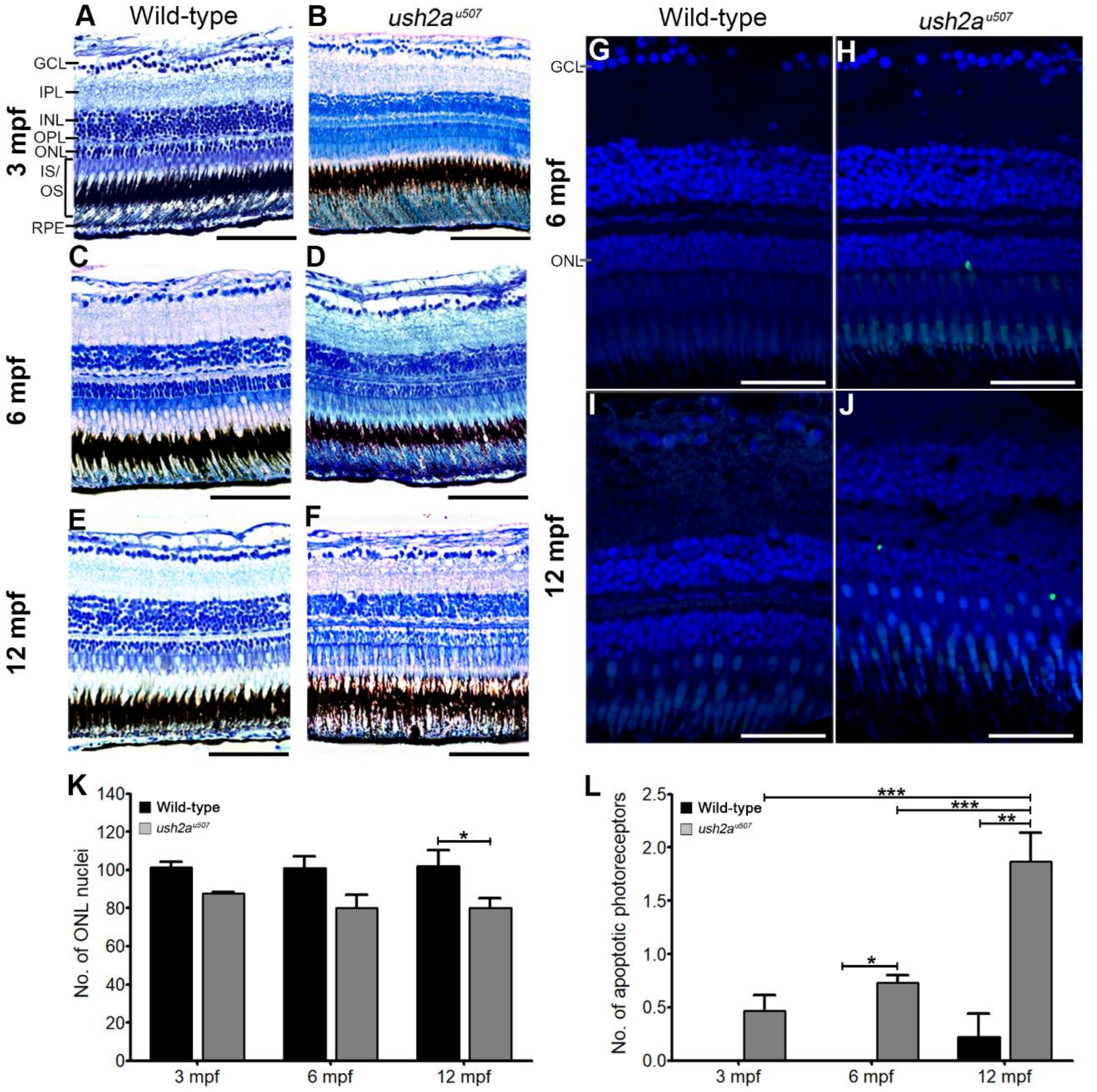

Supplementary Figure S5. Photoreceptor degeneration in the *ush2a*^{u507} retina.
Transverse retinal sections comparing wild-type and *ush2a*^{u507} retinal histology at 3 months post-fertilisation (mpf) (A, B), 6 mpf (C, D) and 12 mpf (E, F). Sections were stained with 1% toluidine blue. TUNEL assays were used to detect apoptotic photoreceptors in the wild-type and *ush2a*^{u507} retinas at 3, 6 and 12 mpf. Representative retinal sections from 6 mpf (G, H) and 12 mpf (I, H) fish are shown. Apoptotic nuclei (green) were detected in the outer nuclear layer (ONL) of the mutant retina at each age. DAPI nucleic acid stain was used as a counterstain (blue). Bar graph (K) shows number of nuclei in ONL per 200 µm at each age (mean ±SEM, n = 3-6). Measurements taken from dorsal and ventral retina immediate to the optic nerve on histology sections. Bar graph (L) shows the number of apoptotic photoreceptors detected per 12 um retinal section at each age (mean ±SEM, n = 9-14). GCL, ganglion cell layer; IPL, inner plexiform layer; INL, inner nuclear layer; OPL, outer plexiform layer; ONL, outer nuclear layer; ON, optic nerve; IS/OS, inner/outer segments; RPE, retinal pigment epithelium. Two-way ANOVA tests were used to test for statistical significance for ONL and apoptotic nuclei, respectively. A significant interaction was found between age and genotype for apoptosis levels ($p = 0.0018$). *$p < 0.05$, **$p < 0.01$, *$p < 0.001$. Scale bars = 50 µm.

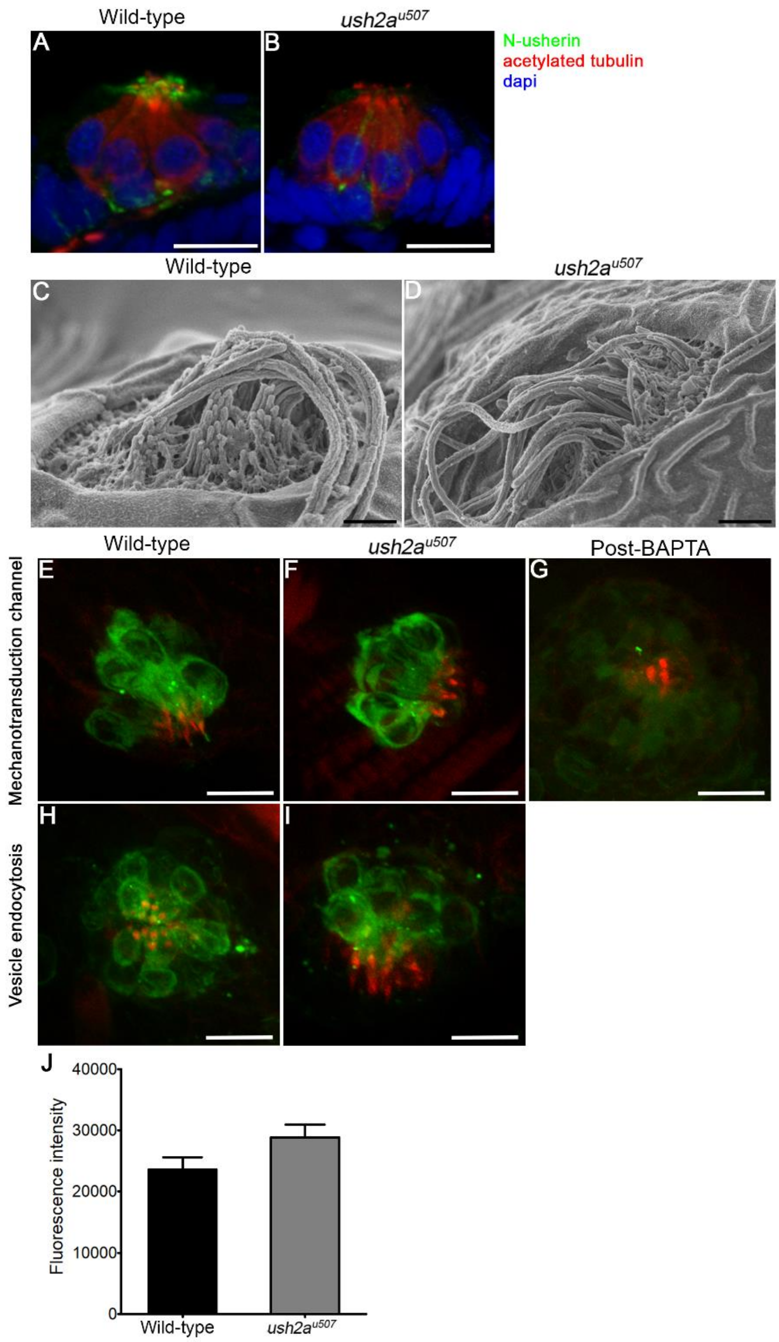

Supplementary Figure S6. Development and function of *ush2a*^{u507} neuromasts.

(A, B) Neuromast cross-sections of 6 days post fertilisation (dpf) wild-type and *ush2a*^{u507} were immunostained with anti-acetylated tubulin (red), anti-usherin-N (green) and DAPI (blue). (C, D) Scanning electron microscopy was used to examine the morphology of the stereociliary hair bundles of 6 dpf wild-type and *ush2a*^{u507} larvae. (E-I) Representative images are shown from 6 dpf neuromasts taken from wild-type and *ush2a*^{u507} zebrafish incubated in FM1-43 dye (green). Phalloidin (red) was used as a counterstain. 30 second FM1-43 incubations were used to assess mechanotransduction channel activity in wild-type (E) and mutant (F) fish, without pre-incubation in BAPTA solution. Wild-type neuromasts incubated in BAPTA before 30 second FM1-43 incubation did not show dye uptake (G), as tip-links were dissolved. For vesicle endocytosis assessment, wild-type and *ush2a*^{u507} zebrafish were pre-incubated in BAPTA before incubation in FM1-43 for 90 minutes (H, I). Bar graph (J) shows fluorescence intensity of FM1-43 per neuromast in wild-type and mutant fish after 90-minute incubation (mean ±SEM). An unpaired t-test was used to test for statistical significance. Scale bar = 10 µm.

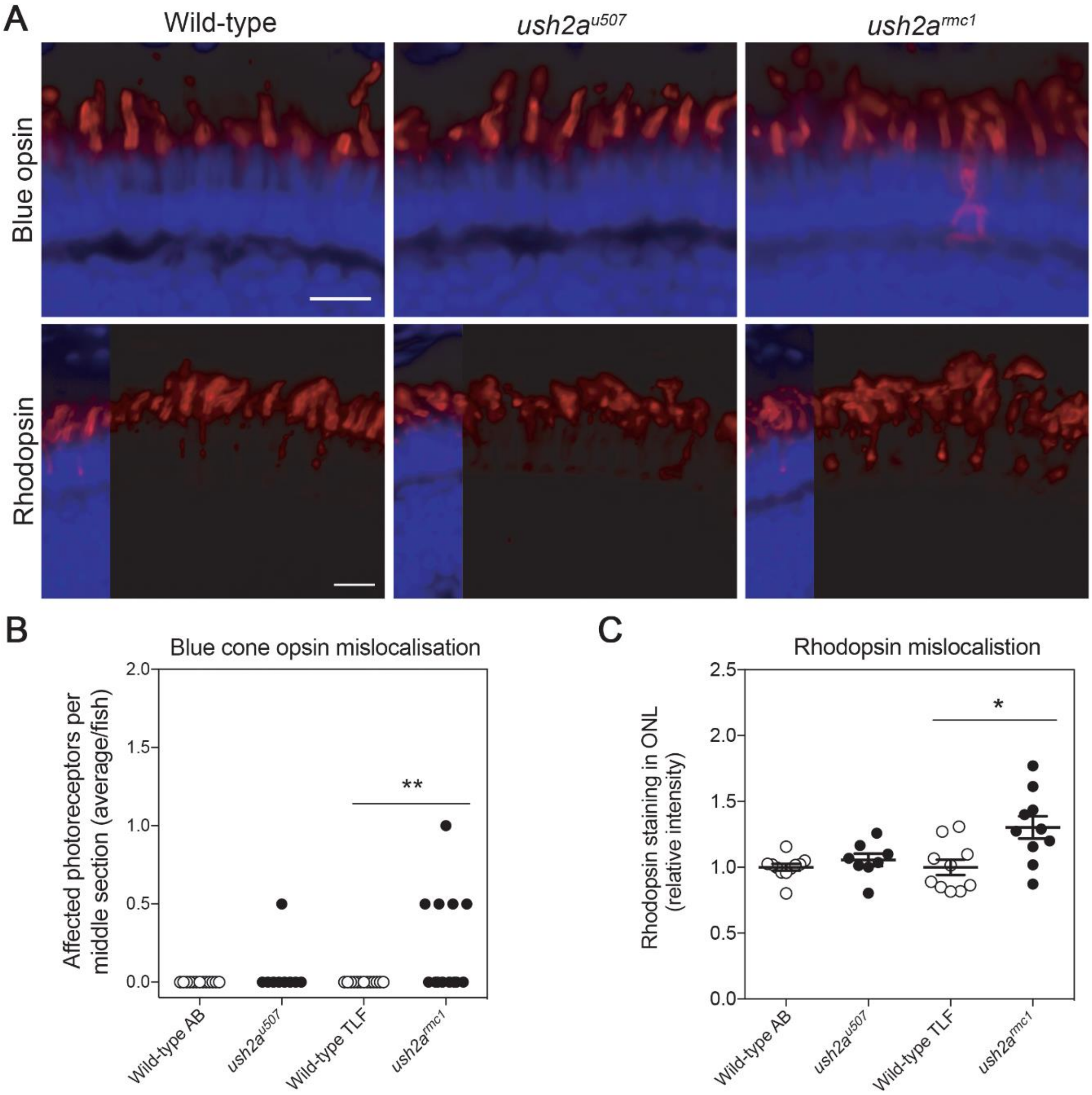

Supplementary Figure S7. Photopigment mislocalisation in *ush2a*^{rmc1} and *ush2a*^{u507} larvae.
(A) Both blue cone opsin and rhodopsin immunoreactivity was mostly restricted to the photoreceptor outer segment in the wild-type and *ush2a*^{u507} retina at 6 dpf. In the *ush2a*^{rmc1} retina, blue cone opsin and rhodopsin was observed occasionally in the inner segment. Images are representative images of each group. (B) Quantification of the number of photoreceptors with blue cone opsin immunoreactivity in the inner segment. For each *ush2a* mutant line, strain-matched wild-type controls were included. (C) Quantification of anti-rhodopsin fluorescent intensity in the photoreceptor inner segment. For each *ush2a* mutant line, strain-matched wild-type controls were included. For both quantifications, a Kruskal-Wallis multiple comparison test, followed by Dunn's post-test, revealed a significant difference in photopigment mislocalisation between the *ush2a*^{rmc1} mutant and appropriate wild-type controls, but not between the *ush2a*^{u507} mutant and appropriate wild-type controls (*$p < 0.05$, **$p < 0.01$). Scale bars = 10 µm.
